# Plant carotenoids: recent advances and future perspectives

**DOI:** 10.1186/s43897-022-00023-2

**Published:** 2022-01-21

**Authors:** Tianhu Sun, Sombir Rao, Xuesong Zhou, Li Li

**Affiliations:** 1 Robert W. Holley Center for Agriculture and Health, USDA-Agricultural Research Service, Cornell University, Ithaca, NY 14853 USA; 2 Plant Breeding and Genetics Section, School of Integrative Plant Science, Cornell University, Ithaca, NY 14853 USA; 3 State Key Laboratory of Crop Genetics & Germplasm Enhancement, Nanjing Agricultural University, Nanjing, 210095 China

**Keywords:** Carotenoids, Metabolism, Accumulation, Regulation, Functional evolution

## Abstract

Carotenoids are isoprenoid metabolites synthesized *de novo* in all photosynthetic organisms. Carotenoids are essential for plants with diverse functions in photosynthesis, photoprotection, pigmentation, phytohormone synthesis, and signaling. They are also critically important for humans as precursors of vitamin A synthesis and as dietary antioxidants. The vital roles of carotenoids to plants and humans have prompted significant progress toward our understanding of carotenoid metabolism and regulation. New regulators and novel roles of carotenoid metabolites are continuously revealed. This review focuses on current status of carotenoid metabolism and highlights recent advances in comprehension of the intrinsic and multi-dimensional regulation of carotenoid accumulation. We also discuss the functional evolution of carotenoids, the agricultural and horticultural application, and some key areas for future research.

## Introduction

Carotenoids are a group of isoprenoid metabolites vital for life. All photosynthetic organisms including plants, algae, and cyanobacteria synthesize carotenoids as indispensable pigments for survival. In plants, carotenoids are essential for photosynthesis and photoprotection. They play critical roles as light harvesting pigments and structural components of photosystems. Carotenoids also provide precursors for the biosynthesis of phytohormones abscisic acid (ABA) and strigolactones (SLs). In addition, carotenoid derivatives can act as signaling molecules in response to environmental and developmental cues or serve as regulators of plant growth. The massive accumulation of carotenoids in many flowers, fruits, and roots contributes to their vivid orange, yellow or red colors and has significant ecological and agronomical value.

Apart from their fundamental roles in plants, carotenoids are also critically important to human nutrition and health. Provitamin A carotenoids, such as β-carotene and α-carotene, are the dietary precursors of vitamin A, which is essential for eyes and immune system. Vitamin A deficiency can cause serious consequences including blindness and death, and affects about a third of preschool children worldwide (https://news.un.org/en/story/2018/05/1008782). Dietary carotenoids as antioxidants help reduce the risk of various chronic diseases such as cancer and cardiovascular diseases (Eggersdorfer and Wyss 2018). In addition, lutein and zeaxanthin as macular pigments are important in decreasing the onset of age-related eye diseases (Sauer et al. 2019). Great efforts have been made to increase carotenoid levels in food crops with enhanced nutritional value and health benefit (Wurtzel et al. 2012; Giuliano 2017; Zheng et al. 2020).

Because of the importance of carotenoids to plants and humans, carotenoid metabolism in plants has been intensively studied (Nisar et al. 2015; Rodriguez-Concepcion et al. 2018; Sun et al. 2018; Wurtzel 2019). The carotenoid biosynthetic pathway is well established and has been widely explored in many plant species. In recent years, great attention has turned to the regulatory control of carotenoid metabolism (Stanley and Yuan 2019; Luan et al. 2020; Sun and Li 2020; Liang et al. 2021). Carotenoid degradation and stable storage have become the other areas of focus since the final carotenoid content in crops is a net result of biosynthesis, turnover, and storage (Cazzonelli and Pogson 2010; Yuan et al. 2015b; Sun et al. 2018; Hermanns et al. 2020; Liang et al. 2021; Torres-Montilla and Rodriguez-Concepcion 2021). While the carotenoid-derived phytohormones ABA and SLs have been extensively investigated (Finkelstein 2013; Chen et al. 2020), the other apocarotenoids in signaling and regulating plant growth and development emerge as an exciting area of study in the carotenoid field (Hou et al. 2016; D'Alessandro and Havaux 2019; Felemban et al. 2019; Moreno et al. 2021). In this review, we focus on current status of carotenoid metabolism and highlight recent advances in our understanding of the intrinsic regulation of carotenoid metabolism at multiple levels. Moreover, we discuss the functional evolution of carotenoids, the agricultural and horticultural application, and the opportunities and directions to further understand carotenoid metabolism and functions in plants.

## Carotenoid metabolism pathway and enzymes

### The core carotenoid biosynthesis pathway in plants

**Fig. 1:**
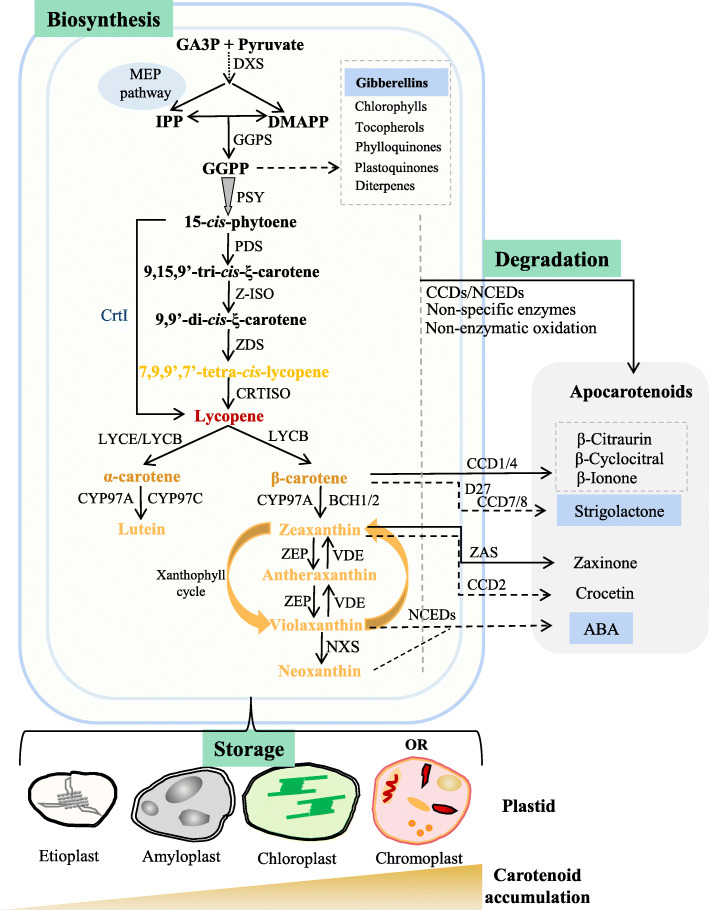
A schematic diagram of plant carotenoid metabolic pathway in plastids. Carotenoid biosynthesis utilizes the plastidial MEP pathway to supply the C5 precursor metabolites IPP and DMAPP. The first committed step in the carotenoid biosynthetic pathway involves the condensation of two C_20_ GGPPs into C_40_ carotenoid phytoene catalyzed by PSY, a major rate-limiting enzyme for carotenoid biosynthesis. Carotenoid degradation involves specific enzymatic oxidative cleavages by CCDs and NCEDs, nonspecific enzymes, and non-enzymatic oxidation to produce diverse apocarotenoids including phytohormones ABA and strigolactones. Phytohormones are highlighted with blue color. Various plastids provide different storage capacity to accumulate very little of carotenoids in etioplasts to massive amounts of carotenoids in chromoplasts. OR is the only known *bona fide* regulator of chromoplast biogenesis. MEP, methylerythritol 4-phosphate; GA3P, glyceraldehyde 3-phosphate; IPP, isopentenyl diphosphate; DMAPP, dimethylallyl diphosphate; GGPP, geranylgeranyl diphosphate; DXS, 1-deoxy-D-xylulose 5-phosphate synthase; GGPPS, GGPP synthase; PSY, phytoene synthase; PDS, phytoene desaturase; Z-ISO, ζ-carotene isomerase; ZDS, ζ-carotene desaturase; CrtISO, carotenoid isomerase; CrtI, bacterial phytoene desaturase; LCYE, lycopene ε-cyclase; LCYB, lycopene β-cyclase; BCH, β-carotene hydroxylase; CYP97A, cytochrome P450 carotene β-hydroxylase; CYP97C, cytochrome P450 carotene ε-hydroxylase; ZEP, zeaxanthin epoxidase; VDE, violaxanthin de-epoxidase; NXS, neoxanthin synthase; CCD, carotenoid cleavage dioxygenase; NCED, 9-*cis*-epoxycarotenoid dioxygenase; ZAS, zaxinone synthase; ABA, abscisic acid; OR, ORANGE protein

Plant carotenoids are mainly tetraterpenoids and synthesized *de novo* in nearly all kinds of plastids (Sun et al. 2018; Li et al., 2016). Carotenoid biosynthesis starts with the synthesis of the basic C5 building blocks of isopentenyl pyrophosphate (IPP) and its allylic isomer dimethylallyl pyrophosphate (DMAPP) via the plastid-localized methylerythritol 4-phosphate (MEP) pathway (Fig. 1). The 1-deoxy-D-xylulose 5-phosphate synthase (DXS) is regarded as the major rate-limiting enzyme in the MEP pathway (Estevez et al. 2001). Sequential condensation of three IPP units to DMAPP generates the C20 precursor geranylgeranyl pyrophosphate (GGPP) via GGPP synthase (GGPPS). Plant genomes typically contain multiple copies of GGPPS with several cell compartments and one or two isoforms appear to be important for the production of most GGPP needed for cell functions (Barja et al. 2021; Barja and Rodriguez-Concepcion 2021). Direct interactions between GGPPS and various GGPP-consuming enzymes allow channeling GGPP for the production of carotenoids, gibberellins, chlorophylls, tocopherols, phylloquinones, plastoquinones or other diterpenes (Ruiz-Sola et al. 2016; Zhou et al. 2017; Barja and Rodriguez-Concepcion 2021) (Fig. 1).

The core carotenoid biosynthesis pathway comprises steps of condensation, desaturation/isomerization, hydroxylation, oxidation, and epoxidation to generate various carotenes and xanthophylls (Nisar et al. 2015; Rodriguez-Concepcion et al. 2018; Sun et al. 2020a). Phytoene synthase (PSY) is the first committed enzyme in the specific carotenoid biosynthesis pathway and catalyzes the condensation of two GGPP molecules to yield the first carotenoid 15-*cis*-phytoene (Fig. 1). PSY is regarded as a major rate-limiting enzyme of carotenoid biosynthesis (Cazzonelli and Pogson 2010; Nisar et al. 2015; Sun et al. 2018). Its activity plays a key role in channeling the metabolic flux into the pathway (Maass et al. 2009; Rodriguez-Villalon et al. 2009) and greatly affects carotenoid content (Paine et al. 2005). *PSY* is commonly present as a small family with up to three members in plant genomes. The PSY isoforms show tissue-specific expression patterns in plants and evolved with different biochemical properties and enzymatic activities (Fraser et al. 2000; Cao et al. 2019). The 15-*cis*-phytoene is then sequentially desaturated and isomerized to produce red colored all-*trans*-lycopene catalyzed by phytoene desaturase (PDS), ζ-carotene isomerase (ZISO), ζ-carotene desaturase (ZDS), and carotenoid isomerase (CRTISO) instead of a single phytoene desaturase CrtI in bacteria (Sandmann 2021). The multi-enzymatic steps in plants enable the production of various *cis*-carotenes with signaling roles in regulating leaf and plastids development (Avendano-Vazquez et al. 2014; Cazzonelli et al. 2020). The subsequent cyclization of all-*trans*-lycopene by lycopene ε-cyclase (LCYE) and/or lycopene β-cyclase (LCYB) leads to the formation of symmetric orange β- and α-carotene in the β-β and β-ε branch, respectively. The molecular synergism between these two bifurcated branches regulates the flux through the branches and affects the downstream carotenoid production (Harjes et al. 2008).

Four carotenoid hydroxylases including two non-heme β-ring hydroxylases (BCH1 and BCH2) and two cytochrome P450 type hydroxylases (CYP97A and CYP97C) catalyze the hydroxylation of α- and β-carotene to produce yellow xanthophylls lutein and zeaxanthin, respectively (Fig. 1). Zeaxanthin in the β-β branch is catalyzed by zeaxanthin epoxidase to yield violaxanthin and reversed back by violaxanthin de-epoxidase (VDE). The interconversion, called xanthophyll cycle, represents a critical machinery to protect plants at high light intensity (Jahns and Holzwarth 2012). The less characterized neoxanthin synthase (NXS) catalyzes the formation of neoxanthin (Neuman et al. 2014; Perreau et al. 2020), which concludes the core biosynthetic pathway (Nisar et al. 2015; Rodriguez-Concepcion et al. 2018; Sun et al. 2020a).

Following the evolution of new enzymes or enzymes with novel functions, an array of species-specific carotenoids are produced, such as capsanthin and capsorubin by a bifunctional enzyme capsanthin/capsorubin synthase (CCS) in red pepper (*Capsicum annuum*) and tiger lily (*Lilium lancifolium*), and astaxanthin in the petals of pheasant’s eye (*Adonis* sp.). The synthesized carotenoids are also modified often by esterification to enhance carotenoid accumulation and stability (Berry et al. 2019; Watkins and Pogson 2020) or by glycosylation to increase fluidity (Diretto et al. 2019).

### Carotenoid Degradation Pathways

Carotenoids as hydrocarbon molecules with conjugated double bonds are unstable. They continuously degrade in cells and sometimes at high rate (Beisel et al. 2010). Both enzymatic and non-enzymatic oxidative cleavages are involved in carotenoid degradation (Fig. 1), which produce diverse apocarotenoids including phytohormones, pigments, volatiles, growth regulators, and signal molecules in plants (Sun et al. 2020a; Liang et al. 2021; Moreno et al. 2021). Carotenoids undergo specific enzymatic oxidative breakdown, which is catalyzed by a family of enzymes named as carotenoid cleavage oxygenase (CCO) (Ahrazem et al. 2016; Beltran and Stange 2016; Liang et al. 2018; Dhar et al. 2020). Plant CCO family consists of 9-*cis*-epoxycarotenoid dioxygenases (NCEDs) and carotenoid cleavage dioxygenases (CCDs). CCDs are classified into several subfamilies CCD1, CCD2, CCD4, CCD7, CCD8, and the newly identified zaxinone synthase (ZAS) 1990 and CCD10 (Wang et al. 2019; Ablazov et al. 2020; Zhong et al. 2020). CCOs cleave carotenoids at specific sites with substrate specificity, therefore dictating the types of apocarotenoid degradation products.

Two groups of CCOs are required for phytohormone ABA and SL synthesis. NCEDs specifically cleave 9-*cis*-violaxanthin and 9-*cis*-neoxanthin to form xanthoxin, the first committed step for ABA production (Schwartz et al. 1997; Tan et al. 2003). CCD7 and CCD8 sequentially cleave 9-*cis*-β-carotene to produce carlactone for SL biosynthesis (Alder et al. 2012). In recent years, the SL biosynthesis pathway has been well elucidated (Jia et al. 2018; Fiorilli et al. 2019; Moreno et al. 2021).

Various other CCDs negatively modulate carotenoid content or are involved in volatile and color formation in plants (Ahrazem et al. 2016; Beltran and Stange 2016; Liang et al. 2018; Dhar et al. 2020). CCD1 is localized outside plastids and cleaves a wide range of carotenoids at various double bond positions (Vogel et al. 2008). Its expression is associated with the production of volatiles and carotenoid level in some cases (Auldridge et al. 2006; Dutta et al. 2021). CCD4 also has broad substrate specificity and affects carotenoid content and pigmentation in various plant species (Ohmiya et al. 2006; Falchi et al. 2013; Gonzalez-Jorge et al. 2013; Zheng et al. 2019; Gao et al. 2021). CCD2 is limited in Crocus species and cleaves zeaxanthin for saffron crocin production (Frusciante et al. 2014; Ahrazem et al. 2016). Recent works identify glycosyltransferases and specific ABC transporters involved in the synthesis and transport of crocins (Demurtas et al. 2019; Diretto et al. 2019; López et al. 2021). CCD10 was identified in some plant species that codes a unique CCD and facilitates maize tolerance to phosphate starvation (Zhong et al. 2020) .

More apocarotenoids with phytohormone-like functions in regulating plant growth and development, symbiosis, and plant defense against herbivores have been unraveled (Moreno et al. 2021). They include molecules of β-cyclocitral, β--cyclogeranic acid, β-ionone, loliolide, and α-ionone from either β-carotene or α-carotene degradation (Wei et al. 2011; D'Alessandro et al. 2018; Dickinson et al. 2019; Murata et al. 2019) as well as a diapocarotenoid anchorene from cleavage of violaxanthin (Jia et al. 2019b). Recent studies identified a new CCD subfamily ZAS, common in most land plants, which cleaves zeaxanthin to produce zaxinone in regulating plant growth as well as strigolactone and ABA biosynthesis (Wang et al. 2019; Ablazov et al. 2020). While some of the apocarotenoids act as signaling molecules, others such as anchorene and zaxinone fulfill their function via interaction with hormones (Moreno et al. 2021).

In addition to the CCD-mediated specific cleavages, carotenoid degradation is also carried out by nonspecific enzymes including lipoxygenases and peroxidases as well as by photochemical oxidation (Sun et al. 2020a). The nonspecific oxidation of carotenoids results in the production of unspecific apocarotenoid products by random cleavage. Recent studies highlight the significant contribution of nonspecific enzyme and non-enzymatic oxidation for the degradation of carotenoids and production of apocarotenoids during fruit maturation and grain post-harvest storage (Schaub et al. 2017; Gao et al. 2019). Further catabolism of many apocarotenoids in plants remains to be fully elucidated. A recent work on the detoxification mechanism of apocarotenoids sheds light on plant apocarotenoid metabolism (Koschmieder et al. 2021).

## Accumulation and storage of carotenoids in plastids

### Plastids and carotenoid accumulation

Plastids are the main site for carotenoid biosynthesis and storage. Different type of plastids has dramatically different ability and capacity to accumulate carotenoids, ranging from very little of carotenoids in etioplasts to massive amounts of carotenoids in chromoplasts (Li et al. 2016; Sun et al. 2018) (Fig. 1). Etioplasts are found in dark-grown tissues accumulating mainly lutein and violaxanthin (Park et al. 2002). Carotenoids along with chlorophyll precursor accumulate in the membranous structure prolamellar body (PLB) to secure the transition into chloroplasts upon illumination (Park et al. 2002; Pipitone et al. 2021). The very low level of carotenoids in etioplasts is likely a consequence of low expression and activity of the rate limiting enzyme PSY suppressed by phytochrome-interacting factors (PIFs) (Toledo-Ortiz et al. 2010) and associated with PLB in an inactive form (Welsch et al. 2000). Similarly, a light-dependent protochlorophyllide oxidoreductase (LPOR) responsible for chlorophyll biosynthesis was recently discovered to be in inactive form as the most abundant protein in the PLB membrane of etioplasts (Floris and Kuhlbrandt 2021). During de-etiolation light activates *PSY* and *LPOR* for the photosynthetic pigment synthesis and together with a *cis*-carotene derived apocarotenoid signal (Cazzonelli et al. 2020) among others to initiate chloroplast development from etioplasts.

Amyloplasts store starch granules in seeds, roots, and tubers. Amyloplasts primarily accumulate various xanthophylls such lutein, zeaxanthin, and violaxanthin in the envelope membranes (Lopez et al. 2008a). As starch-storing plastids, amyloplasts generally accumulate limited amounts of carotenoids (Wurtzel et al. 2012). A number of factors such as low biosynthetic capacity, lack of lipoprotein sequestering substructures, and carbon flux primarily toward starch synthesis may all restrict carotenoid biosynthesis, accumulation, and/or stable storage in amyloplasts (Li et al. 2016; Sun et al. 2018). However, amyloplasts have the potential to accumulate relatively high levels of carotenoids as documented in many transgenic studies (Paine et al. 2005; Diretto et al. 2007; Bai et al. 2016; Mortimer et al. 2016). Because many starchy crops are low in carotenoid content, it is greatly important to enrich and stably store carotenoids particularly provitamin A carotenoids in those crops for improving human nutrition and health (Giuliano 2017; Sun et al. 2018; Zheng et al. 2020).

Chloroplasts are the site of photosynthesis. Carotenoids play indispensable roles in photosynthesis and photoprotection. Carotenoids predominantly as lutein, β-carotene, violaxanthin, and neoxanthin accumulate in relatively high abundance in chloroplasts but the color is normally masked by chlorophylls. Thylakoid membranes and the light harvesting complexes are the main sites to harbor carotenoid molecules (Ruiz-Sola and Rodriguez-Concepcion 2012), but how carotenoids are delivered to these sites from the biosynthetic location of mainly envelopes remains unknown. Recently, a chloroplast Sec14-like 1 (CPSFL1) protein was reported to bind and transport carotenoids in *Chlamydomonas* (García-Cerdán et al. 2020). This study brings out the possibility of translocation of carotenoid metabolites in chloroplasts.

Chromoplasts are the main site to store diverse and large amounts of carotenoids in many colorful organs of horticultural crops (Egea et al. 2010; Li and Yuan 2013; Yuan et al. 2015b; Sun et al. 2018; Ohmiya et al. 2019; Sadali et al. 2019; Hermanns et al. 2020). Chromoplasts can be derived from chloroplasts during ripening processes such as in tomatoes, and also arise from proplastids and amyloplasts in non-photosynthetic tissues (Li et al. 2001; Horner et al. 2007; Egea et al. 2011). Chromoplasts harbor carotenoid sequestration substructures, which are diverse in different species and tissues and sometimes even coexist in the same tissues (Schweiggert and Carle 2017). The diversity of those sequestration substructures including globular, crystalline, membranous, fibrillar, and tubular type is likely contributed by the level and kind of carotenoids accumulated or vice versa (Hermanns et al. 2020; Wen et al. 2020).

### Genes that regulate chromoplast formation

Although chromoplasts are frequently observed in many vegetables and fruits, the genes that control chromoplast biogenesis and duplication are less known. The *ORANGE (OR)* gene represents the only known *bona fide* regulator of chromoplast biogenesis. The gain-of-function alleles of *OR* are responsible for high β-carotene accumulation in orange curd cauliflower and melon fruit (Lu et al. 2006; Tzuri et al. 2015) as well as apparently in carrot and sweetpotato (Ellison et al. 2018; Gemenet et al. 2020; Coe et al. 2021). Although wild type OR regulates PSY protein stability (Zhou et al. 2015b; Park et al. 2016; Welsch et al. 2018), the high level of carotenoid accumulation in the *OR* mutants is not due to the biosynthetic activity (Li et al. 2001; Li et al. 2006; Chayut et al. 2015; Chayut et al. 2017). Instead, it is the direct consequence of chromoplast biogenesis (Lu et al. 2006; Lopez et al. 2008b; Li et al. 2012; Yuan et al. 2015a; Chayut et al. 2017; Yazdani et al. 2019). Co-expression of *PSY* and the *OR* gain-of-function allele to initiate chromoplast biogenesis dramatically enhances provitamin A and total carotenoid content and stability in Arabidopsis seeds (Sun et al. 2021), showing the effectiveness of regulating chromoplast storage sink formation along with biosynthetic activity for carotenoid enrichment and stable storage in seeds.

Chromoplast number and size in a cell are critically important for its capacity to synthesize and store carotenoids. Recently, it was discovered that chromoplast duplication employs the binary division machinery as chloroplasts (Sun et al. 2020b). While the gain-of-function alleles of *OR* promote chromoplast biogenesis, only one or two large chromoplasts are present in each affected cell (Li et al. 2001; Chayut et al. 2017). The natural variant of OR found in melon, OR^His^, was discovered to specifically interact with Accumulation and Replication of Chloroplasts 3 (ARC3) and compete with Paralog of ARC6 (PARC6) in suppressing chromoplast duplication (Sun et al. 2020b). Such a suppression and restriction can be relaxed by increasing the expression of other plastid division factors such as Plastid Division 1 (PDV1), which leads to increased number of chromoplasts in the *OR*^*His*^ plant (Sun et al. 2020b). Moreover, wild type OR was found to mediate chloroplast biogenesis in etiolated Arabidopsis cotyledons via interacting with the transcription factor TCP14 (Sun et al. 2019) and regulate preprotein import through interacting with several Translocons at the Inner-envelope Membranes of Chloroplasts (TIC) in facilitating the late stage of plastid pre-protein translocation (Yuan et al. 2021). OR represents a multifunctional regulator in plastid development (Zhou et al. 2011; D'Andrea et al. 2018; Sun et al. 2019; Chayut et al. 2021; Chen et al. 2021; Kim et al. 2021) in addition to carotenoid biosynthesis and accumulation (Kim et al. 2018; Feder et al. 2019; Osorio 2019; Miyagishima 2020).

Transcriptional regulators have been identified to affect carotenoid accumulation in chromoplasts but most factors are also general regulators associated with fruit ripening (Stanley and Yuan 2019; Sun and Li 2020). RCP2, a tetratricopeptide repeat protein, was recently shown to be sufficient in regulating chromoplast development for carotenoid accumulation in monkeyflowers (Stanley et al. 2020). However, whether it is a developmental-associated or chromoplast-specific regulator needs to be further explored. Recently, it was reported that loss of photosynthetic competence and enhanced carotenoid accumulation in leaf tissue elicits chloroplast to chromoplast transition (Llorente et al. 2020), revealing a mechanistic basis for chromoplast formation. Moreover, ubiquitin E3 ligase SP1 homologues in the chloroplast-associated protein degradation proteolytic pathway were shown to promote chloroplast to chromoplast transition through reconfiguration of the plastid protein import machinery (Ling et al. 2021). While the knowledge underlying chromoplast formation is increasing during the past years (Torres-Montilla and Rodriguez-Concepcion 2021), clearly the nature of chromoplast biogenesis for high levels of carotenoid accumulation needs to be further explored.

## Hierarchical regulation of carotenoid metabolism

**Fig. 2:**
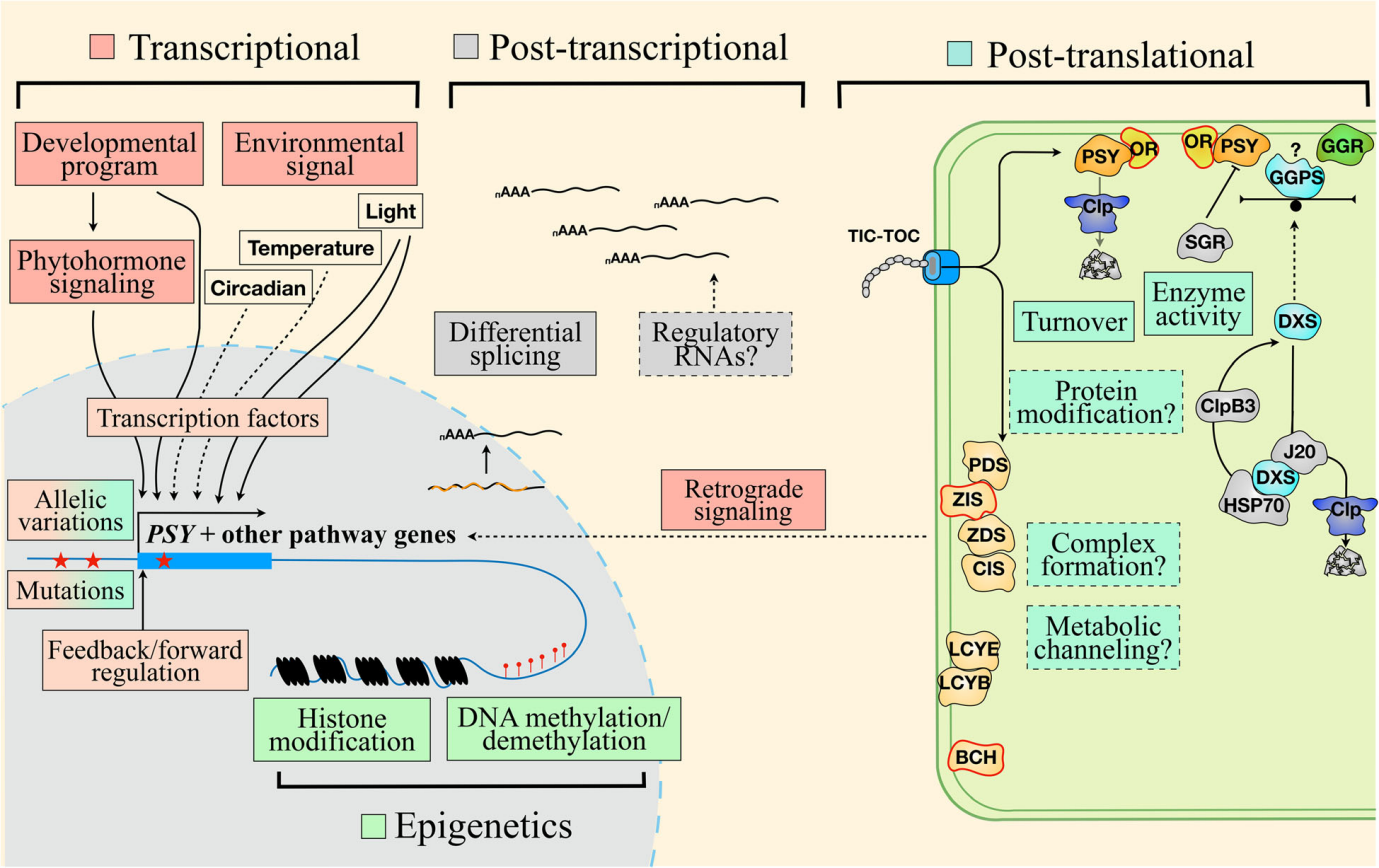
Hierarchical regulation of carotenoid metabolism. Carotenoid metabolism is regulated by many signals including environmental cues, developmental program, and phytohormone signaling along with retrograde signaling. These signals converge at the level of transcription factors to regulate carotenogenic gene expression. In addition, allelic variation, mutations, and feedback and forward regulation affect the transcription of pathway genes or the activities of key enzymes. Epigenetic regulation also controls carotenogenic gene expression. Differential splicing provides a post-transcriptional regulation of key carotenogenic genes. Post-translational regulation plays a critical role in the control of metabolic pathway activity and fine-tune carotenoid production through mechanisms including protein-protein interaction, enzyme complex formation, metabolic channeling, and potential protein modification

Being essential to the plant's life in green tissues and accumulating in diverse amounts and composition in other organs of crops, carotenoids are synthesized under tight regulation and fine-tuning in response to the environmental and developmental cues. Significant progress has been made in our understanding of the regulatory mechanisms underlying carotenoid biosynthesis. Multiple layers of regulation including transcriptional, posttranscriptional and post-translational regulation, and epigenetic control are involved in modulating the pathway activity (Ruiz-Sola and Rodriguez-Concepcion 2012; Luan et al. 2020; Sun and Li 2020), which are depicted in Figure 2. Information is also emerging for the regulation of carotenoid degradation (Watkins and Pogson 2020; Liang et al. 2021). Investigation of carotenogenic regulatory mechanisms underlines the complexity of crosslinking with other cellular processes, which will not be reviewed here.

### Transcriptional regulation

Transcriptional regulation has long been the focus in understanding of the regulatory control of carotenogenesis. Transcriptional regulation represents the first and primary regulatory mechanism of carotenoid biosynthesis (Ruiz-Sola and Rodriguez-Concepcion 2012; Sun and Li 2020). It is the major contributor to the huge diversity of carotenoids in horticultural crops (Yuan et al. 2015b; Ohmiya et al. 2019; Hermanns et al. 2020). Modulation of the pathway structural gene expression such as by transcription factors, mutations, natural variations, and feedback and feedforward can all affect carotenogenesis in plants (Fig. 2).

**Fig. 3:**
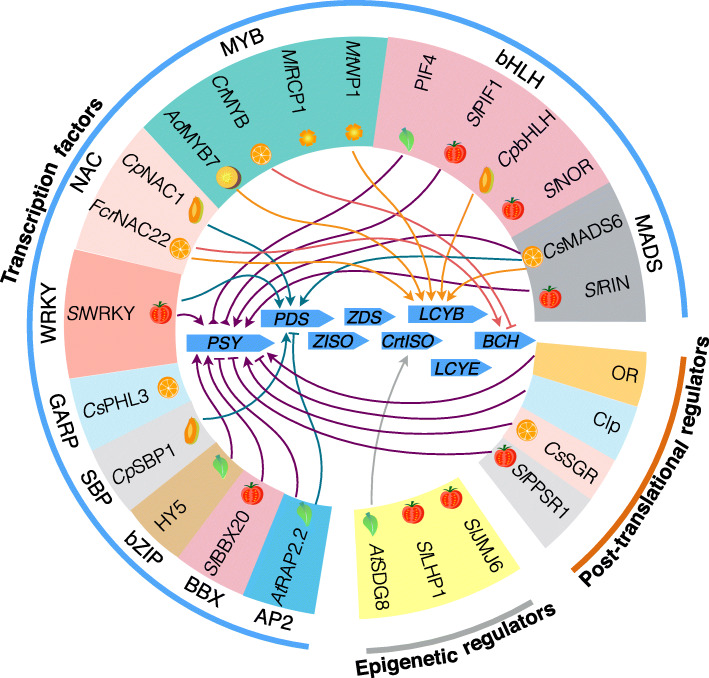
Summary of some known transcription factors, post-translational regulators, and epigenetic regulators that regulate the expression of carotenogenic pathway genes or PSY protein. Their direct actions on pathway genes or PSY protein in different organs such as leaf, carotenoid-rich fruit, and flower are indicated. Those transcription factors are clustered by the family of transcription factors. MYB, Myeloblastosis family of transcription factors; RCP1, REDUCED CAROTENOID PIGMENTATION1; WP1, WHITE PETAL1; NAC, NAM, ATAF, and CUC transcription factors; WRKY, WRKYGQK motif-containing transcription factors; PHL3, PHOSPHATE STARVATION RESPONSE FACTOR LIKE3; SBP1, SQUAMOSA PROMOTER BINDING PROTEIN1; HY5, ELONGATED HYPOCOTYL5; BBX, B-box transcription factors; RAP2.2, APETALA2/ethylene response factor-type transcription factor 2.2; PIF, PHYTOCHROME-INTERACTING FACTORS; bHLH, basic Helix-Loop-Helix transcription factors; NOR, NON-RIPENING transcription factor; MADS, MADS-box containing transcription factors; RIN, RIPENING INHIBITOR; OR, ORANGE protein; Clp, Clp protease; SGR, STAY GREEN; PPSR1, Plastid Protein Sensing RING E3 ligase 1; JMJ6, Jumonji C-terminal (JmjC) domain-containing demethylase 6; LHP1, LIKE HETEROCHROMATIN PROTEIN1; SDG8, SET DOMAIN GROUP 8. At, *Arabidopsis thaliana*; Sl, *Solanum lycopersicum*, tomato; Cp, *Carica papaya* L., papaya; Cs, *Citrus sinensis*, sweet orange; Cr, *Citrus reticulate*, mandarin orange; Ad, *Actinidia deliciosa*, kiwifruit; Ml, *Mimulus lewisii*, monkeyflower; Mt, *Medicago truncatula*

Transcription factors are central in regulating transcription of carotenogenic genes. Recent advances have identified and validated transcription factors or regulators that transcriptionally activate or suppress the expression of carotenoid structural genes directly in plant species as reviewed (Stanley and Yuan 2019; Hermanns et al. 2020; Sun and Li 2020). Additional carotenogenic transcription factors have been continuously identified (Meng et al. 2020; Gong et al. 2021; Lu et al. 2021b; Zhu et al. 2021a). These transcription factors transcriptionally regulate single or multiple pathway genes (Fig. 3). While the investigated transcription factors have been shown to directly bind to the promoters of carotenoid metabolic pathway genes, whether they are *bona fide* regulators and function across or within the same crop species to modulate carotenoid metabolism remains to be fully documented. In addition, many transcription factors are general regulators involved in multiple processes of plant growth and development. Some likely work indirectly to affect carotenoid metabolism (Stanley and Yuan 2019).

Environmental and developmental signals are known to regulate carotenoid metabolism (Dhami and Cazzonelli 2020; Sun and Li 2020) (Fig. 2). However, how these signals converge at the level of transcription factors is not well understood. Light is the most important cue to regulate carotenoid structural gene expression in photosynthetic tissues and in carrot roots (Llorente et al. 2017; Quian-Ulloa and Stange 2021). PIFs and ELONGATED HYPOCOTYL5 (HY5), the key antagonistic regulators of photomorphogenesis, directly regulate *PSY* during de-etiolation and in response to shade and temperature, linking light signaling with *PSY* transcription (Toledo-Ortiz et al. 2010; Toledo-Ortiz et al. 2014; Bou-Torrent et al. 2015; Wang et al. 2021b). Interestingly, a number of photomorphogenesis and light reception genes appear also regulating carotenoid biosynthesis and carrot storage root development in dark (Quian-Ulloa and Stange 2021). FcrNAC22 was recently shown to mediate the red light-induced carotenogenesis in kumquat fruit (Gong et al. 2021). Moreover, light triggers photoisomerization of *cis*-carotenes to affect carotenogenesis (Park et al. 2002; Cazzonelli et al. 2020). In addition to light, other environmental factors such as temperature, circadian, and nutrient status may also affect carotenoid accumulation (Dhami and Cazzonelli 2020). A citrus phosphate starvation response factor CsPHL3 directly binds to the *LCYB1* promoter to negatively regulate carotenoid metabolism, establishing a link with plant nutrient status (Lu et al. 2021a). However, much remains unknown for the environmental signals in activating carotenogenic gene expression.

Developmental signals appear to be the primary determinant of transcriptional regulation of carotenoid genes in fruits and flowers (Sun and Li 2020). In many cases, carotenoid biosynthesis is linked to the transcriptional upregulation of several upstream biosynthetic genes and downregulation of downstream genes (Ronen et al. 1999; Chayut et al. 2015) or altered expression of a few key genes like *PSY1* and *DXS* in chilli pepper (Berry et al. 2019), *LCYB* and *BCH* in red fleshed papaya (Zhou et al. 2019), and *LCYB* in kiwifruit (Ampomah-Dwamena et al. 2019). During flower development, several transcription factors have been identified to regulate carotenoid pigments, such as MYB activators WHITE PETAL1 in *M. truncatula* and reduced carotenoid pigmentation 1 (RCP1) in monkeyflower (Sagawa et al. 2016; Meng et al. 2019). RIN as the master regulator of fruit ripening in tomato directly regulates *SlPSY1* expression (Martel et al. 2011). These studies provide a link between developmental signals and carotenogenic gene expression. Developmental signals also modulate the production of phytohormones. A complex network integrating both developmental and phytohormone signals mediates carotenogenesis during fruit ripening (Liu et al. 2015; Sun and Li 2020). However, many elements in the developmental signaling pathway are still missing.

In addition, mutations in structural genes and regulators can affect their expression at mRNA and/or protein levels to cause the accumulation of specific carotenoids in plants, particularly in horticultural crops. A classic example is tomato fruit with various colors. The yellow, tangerine, orange, and orange-red tomato fruit with the accumulation of different major carotenoids result from mutations in the pathway genes including *PSY1, CrtISO, LCYE*, or *CYCB* (Fray and Grierson 1993; Ronen et al. 1999; Ronen et al. 2000; Isaacson et al. 2002). A missense mutation of *LCYB* that affects the enzyme protein level appears to be responsible for lycopene accumulation in watermelon (Zhang et al. 2020). Mutations in *Z-ISO* or *ZEP* produce yellow and orange fruit in ‘Pinalate’ sweet orange (*Citrus sinensis*), melon, Chinese cabbage, and pepper (Galpaz et al. 2013; Zhang et al. 2015; Rodrigo et al. 2019; Lee et al. 2021).

Natural variations in some key structural or regulatory genes also affect carotenogenic pathway activity and the accumulation of carotenoid final products. Well-known examples are the natural variations of *LYCE* and β-carotene hydroxylase (*crtRB1*) that affect β-carotene level in maize grain (Harjes et al. 2008; Yan et al. 2010) as well as in sweet corn (Baseggio et al. 2020) .The color variation in red chilli pepper is associated with the variations of *DXS* and *PSY1* along capsanthin esteration (Berry et al. 2019). The allelic variations of structural genes alter their transcript abundance/activity and modulate the accumulation of specific carotenoids. Natural variation in the promoter of *CCD4b1* was found to be tightly associated with differential *CCD4b* expression, β-citraurin accumulation and color variation in the citrus peel (Zheng et al. 2019).

Furthermore, feedback and feedforward regulation also provides another control. By investigating lines with a bacterial desaturase (*crtI*) overexpressed in the *tangerine* and *old gold crimson* mutants of tomato, which are defective in *CRTISO* and chromoplast-specific lycopene cyclase *CYCB*, respectively, it was found that altered metabolites cause a negative feedback regulation of *PSY1* and feedforward control of *CYCB* expression (Enfissi et al. 2017). Feedback loops are an important component of regulation in metabolic pathways. A close circuit that senses, connects and coordinates the accumulation of end products with the initial transcriptional and posttranscriptional mechanisms forms the basis of the feedback regulations and maintain the flux to ensure carotenoid homeostasis in plants (Kachanovsky et al. 2012; Fantini et al. 2013; Arango et al. 2014; Wright et al. 2014; Simpson et al. 2018; Koschmieder et al. 2021).

### Post-translational regulation

In comparison with the understanding of transcriptional regulatory mechanisms, less is known about the post-transcriptional and post-translational regulation of carotenogenesis in plants. Carotenoid production in living cells is a dynamic process in responding to various signals and stimuli. Post-translational along with post-transcriptional regulation such as differential splicing (Alvarez et al., 2016) or regulatory RNAs provides additional layer of regulation to modulate carotenogenic enzyme activity and fine-tune carotenoid production (Sun and Li 2020) (Fig. 2). Post-translational regulation includes machineries like protein-protein interactions and metabolic channeling through multi-enzyme complexes (Ruiz-Sola and Rodriguez-Concepcion 2012; Nisar et al. 2015; Sun and Li 2020).

An increasing knowledge of post-translational regulation of carotenogenic enzymes via protein-protein interactions has emerged in recent years. The interactions of carotenogenic enzymes with molecular chaperones and Clp protease adjust the functional forms of pathway enzymes and tightly control their proteostasis for carotenoid biosynthesis. For example, DXS enzyme activity and proteostasis are regulated posttranslationally in response to the physiological status of the plastids. Inactive forms of DXS is recognized by a DnaJ-like protein J20 and delivered to Hsp70 chaperone, which interacts with Hsp100/ClpC1 for degradation via the Clp protease complex and with Hsp100/ClpB3 for activation (Pulido et al. 2013; Pulido et al. 2016; Llamas et al. 2017). Similarly, PSY enzyme protein level and proteostasis are mediated posttranslationally to fine-tune carotenogenesis. PSY physically associates with OR chaperones for activity and with Clp protease recognized by Hsp100/ClpC1 for degradation (Li et al. 2012; Zhou et al. 2015b; Park et al. 2016; Chayut et al. 2017; D'Andrea et al. 2018; Welsch et al. 2018). Interestingly, a recent study reports that a plastid protein sensing RING E3 ligase 1 (PPSR1) interacts with PSY1 and presumably ubiquitinates the PSY1 precursor in cytosol to affect the steady state level of plastidial PSY1 protein for carotenogenesis in tomato fruit (Wang et al. 2020b). It is also discovered that in tomato and citrus, the activity of PSY is regulated by its interaction partner STAY GREEN, a magnesium dechelatase involved in chlorophyll degradation (Luo et al. 2013; Zhu et al. 2021b) (Fig. 3).

Enzyme complex formation is important in affecting the metabolic flux and possibly sub-organellar localization. By enzyme fusions of GGPPS with PSY or 3, 3ˈ β-carotene hydroxylase with 4, 4ˈ β-carotene oxygenase, the metabolic flux is effectively directed toward carotenogenesis and astaxanthin production, respectively (Camagna et al. 2019; Nogueira et al. 2019), implying the importance of enzyme complex in facilitating carotenoid biosynthesis. Although multiple putative complexes are proposed (Ruiz-Sola and Rodriguez-Concepcion 2012), the *in vivo* evidence of the existence of enzyme complexes in carotenogenesis are merely reported except a few enzymes that were shown to be in large protein complexes (Lopez et al. 2008a).

Post-translational modification (PTM) enables a quick regulation of protein function in response to metabolic and environmental changes. Therefore, it is a ubiquitous mechanism for protein activity modification. There are many identified types of PTM in chloroplasts including phosphorylation, lysine acetylation, lysine methylation, tyrosine nitration, S-nitrosylation, glutathionylation, sumoylation, and glycosylation, while phosphorylation is a well-studied post-translational modification of many photosystem proteins (Grabsztunowicz et al. 2017). PTMs can function in regulating some isoprenoid precursor biosynthetic enzymes (Hemmerlin 2013). Several carotenoid biosynthetic enzymes also have predicted phosphorylation sites by PhosPhAt (Durek et al. 2010). However, these PTMs and their roles on carotenogenesis still need to be experimentally identified in the future.

### Epigenetic regulation of carotenogenesis

The discovery of epigenetic regulation of carotenogenesis expands our understanding of the regulatory control of carotenoid metabolism, although it is still a less studied area of carotenoid research. Different epigenetic mechanisms such as histone modifications and DNA methylation and demethylation add another tier of regulation on carotenogenesis (Anwar et al. 2021) (Fig. 2). A well-known regulator of carotenogenesis by histone modifications is SDG8, a histone lysine methyltransferase that specifically regulates the expression of *CrtISO* by maintaining the histone H3 lysine K4 trimethylation (H3K4me3) marks in *CRTISO* promoter and gene body (Cazzonelli et al. 2009). Other histone modifiers including a histone lysine demethylase JMJ6, heterochromatin Protein 1b (LHP1), and the histone variant H2A.Z were found to regulate *PSY1* and/or other carotenogenic gene expression during tomato fruit ripening (Li et al. 2020; Liang et al. 2020; Yuan et al. 2021) (Fig. 3).

DNA methylation and demethylation have been shown to directly or indirectly alter carotenoid levels. As DNA demethylation controls fruit ripening (Zhong et al. 2013; Lang et al. 2017), carotenogenic regulation may represent one of the impacted processes during fruit ripening. TAGL1 is a tomato fruit ripening regulator and its intensive DNA methylation decreases *PSY1* expression and reduces carotenogenesis (Liu et al. 2020). Mutations in demethylases such as *sldml2* result in higher DNA methylation in the promoters of *PSY1, Z-ISO, ZDS*, and *CrtISO* and reduce ripening process including carotenogenesis (Lang et al. 2017). DNA methylation alters the binding of R2R3-MYB transcription factors on target genes (Wang et al. 2020a). Hypomethylation of genomic regions surrounding the transcription start sites of the *CaPSY1, CaPDS, CaRIN* and *CaNCED1* has been shown to regulate their transcript abundance to affect carotenogenesis during pepper fruit ripening (Xiao et al. 2020).

## Functional evolution of carotenoids

### Carotenoids in photosynthesis: accessory or central role?

**Fig. 4:**
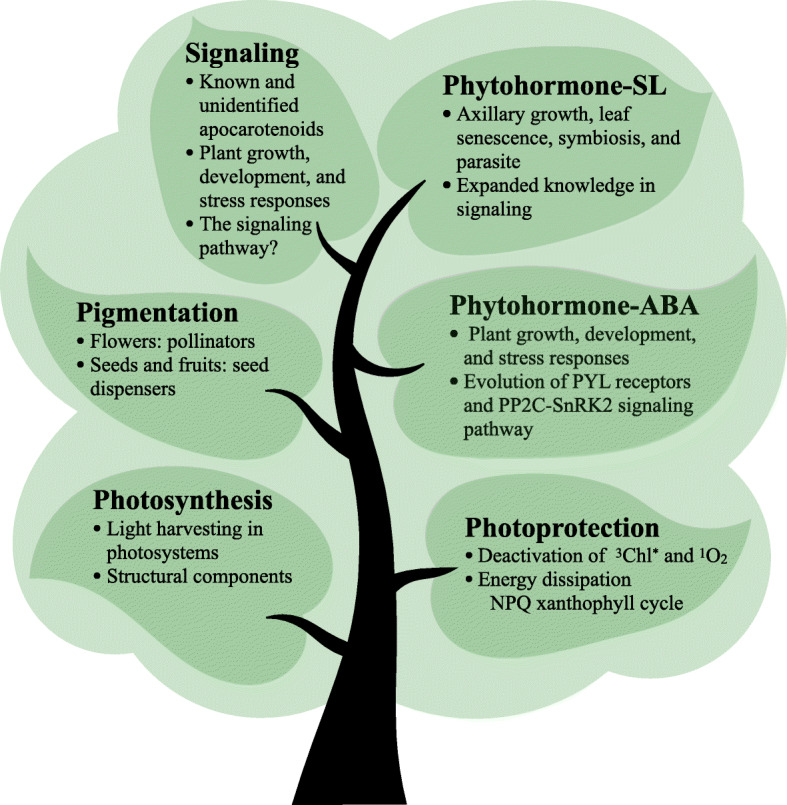
Basic and acquired functions of carotenoids during plant evolution. Carotenoids play central roles in photosynthesis and photoprotection as the key components of photosystems. Carotenoids also contribute to the pigmentation of seeds, fruits, and flowers, which showed co-evolution with seed dispensers and pollinators. Carotenoid derived phytohormone, including abscisic acid (ABA) and strigolactone (SL), arise from the evolution of land plants. Apocarotenoid signaling affects plant growth, development, and stress response, but more signaling molecules and the pathways need to be further elucidated

Carotenoid biosynthesis was coevolved with photosynthesis to provide metabolites with specific functional roles (Takaichi 2011; Sandmann 2021) (Fig. 4). Carotenoids absorb light energy and transfer to chlorophylls in the spectrum 450-550 nm, a range that chlorophylls do not absorb (Hashimoto et al. 2016). As such, carotenoids expand the light wavelength range of photosynthesis for phototrophic organisms including plants and algae.

From an evolutionary aspect, carotenoids have been present in the reaction center in association with bacteriochlorophyll from ancient photosynthetic bacteria (Cardona et al. 2019). During the evolution of different phylogenetic groups, the lateral transfer of carotenoid biosynthetic genes established new connections between carotenoids and photosynthesis (Sandmann 2021). In cyanobacteria, CrtP and CrtQ, which function in phytoene and ζ-carotene desaturations, respectively, started to use oxidized plastoquinones as cofactors in the desaturation reactions (Breitenbach et al. 2013). This change in the carotenoid biosynthesis pathway connects the carotenoid desaturation steps to the photosynthetic electron transport, providing a short-term adaptation mechanism for carotenogenesis to photosynthesis.

In land plants, lutein, β-carotene, violaxanthin and neoxanthin are typical and also the most abundant carotenoids in chloroplasts (Al-Babili and Beyer 2005; Ruiz-Sola and Rodriguez-Concepcion 2012). The light-harvesting complexes (LHCs) as the major light energy collectors in green plants are the assembly of chlorophylls, carotenoids and proteins (Liu et al. 2004; Pan et al. 2011; Pan et al. 2020). Carotenoids play an additional role of structural stabilization in LHCs. Since the energy transport and conversion finally involve chlorophylls, they are traditionally considered playing the leading role while carotenoids are ‘accessory’ pigments (Collini 2019).

The timescale of energy transfer between carotenoids and chlorophylls can be as fast as tens of femtoseconds (Meneghin et al. 2018; Son et al. 2020). The complexity of the light harvesting apparatus also brings challenges to reveal the intrinsic role of carotenoids in photosynthesis. In recent years, with the application of structural biology and ultrafast detection technology, the role of carotenoids is reconsidered from ‘accessory’ to ‘central’ (Collini 2019). With the quantum chemical evaluation, the carotenoid to chlorophyll energy transfer was found to be coherent rather than loose interaction (Ghosh et al. 2017; Meneghin et al. 2018). The lutein molecules in LHCII have been identified as key chromophores for the control of the excitation energy flow (Son et al. 2019). These studies point a central role of carotenoids rather than accessory function in light harvesting.

Besides being photosynthetic complex-bound, carotenoids also perform a structural role in the formation and dynamics of the thylakoid membrane system and regulate thylakoid membrane fluidity (Havaux and Gruszecki 1993; Havaux 1998; Bykowski et al. 2021). Moreover, xanthophyll carotenoids were found to function as “glue” molecules to stabilize chlorophyll biosynthesis in cyanobacteria (Proctor et al. 2020). Since cyanobacteria are the prokaryotic origin of chloroplast, such a function of carotenoids may occur in chloroplasts, which needs to be further investigated.

### Photoprotection: guarding the plants

Carotenoids have a vital function in photoprotection to adapt to the changing light environment. They are known to deactivate triplet chlorophyll and singlet oxygen as well as to dissipate excess light energy to prevent photodamage of the photosynthetic apparatus in LHCs of photosystem II (Frank and Cogdell 1996; Jahns and Holzwarth 2012) (Fig. 4). β-Carotene is present in the core of photosystems in all organisms and quenches singlet oxygen (Telfer 2005; Umena et al. 2011; Qin et al. 2015), although a recent study shows that other carotenoids can replace it (Xu et al. 2020). Xanthophylls are present in the peripheral LHC (Qin et al. 2015). There have been tremendous evolutionary changes in the antenna systems that not only capture and transfer light energy but also dissipate excitation energy in land plants (Ruban and Murchie 2012).

As the most rapid and efficient mechanism of excess light energy dissipation into heat from photosystem II, non-photochemical chlorophyll fluorescence quenching (NPQ) involves the conversion of violaxanthin into zeaxanthin (Niyogi and Truong 2013; Murchie and Ruban 2020). The cyclical interconversion of violaxanthin, antheraxanthin and zeaxanthin, also known as xanthophyll cycle (Fig. 1), exists in green algae and land plants as an energy dissipation strategy and thereby reducing reactive oxygen species production (Jahns and Holzwarth 2012). In land plants, zeaxanthin plays a central role in NPQ because quenching of excess absorbed light energy undergoes energy transfer from chlorophylls to chlorophyll-zeaxanthin heterodimer before being dissipated into heat (Holt et al. 2005; Ahn et al. 2008). The presence of zeaxanthin has been shown to influence the interaction of the antenna system LHCII with PsbS, essential in NPQ for photoprotection (Wilk et al. 2013; Sacharz et al. 2017).

### Pigmentation: coevolution with pollinators and seed dispensers

Many flowering plants rely on pollinators to facilitate the reproductive process. The interactions between flower and pollinator are a major driver for floral trait diversification and speciation (Kay and Sargent 2009; Yuan et al. 2013). Pigmentation is an important trait to attract specific pollinators and natural pollinators have clear preferences to flower pigmentations (Shang et al. 2011). In the model system *Mimulus lewisii*, the lower flower petals contain two yellow ridges pigmented by carotenoids, which are regulated by RCP1 and RCP2 (Sagawa et al. 2016; Stanley et al. 2020). This pigmentation pattern specifically serves as nectar guides for the bumblebee pollinators (Owen and Bradshaw 2011). A recent study of floral traits in bee and hummingbird pollinated sister species of *Aquilegia* further suggests the importance of evolving suites of traits including carotenoid color trait with pollinators (Edwards et al. 2021). The combination of biotechnology and evolutionary genomics provides essential methods to understand the evolutionary dynamics of flower pigmentation and pollinator attraction mechanisms (Frachon et al. 2021).

Some seeds accumulate high levels of vivid colored carotenoids, which are visually attractive to seed dispensers like birds. For example, the red color carotenoid tobiraxanthins in *Pittosporum tobira* seeds act as attractant for birds to eat seeds and disperse them (Fujiwara et al. 2002). Carotenoids contribute to the bright color of fruits, which is a conspicuous visual signal to encourage discovery and consumption by seed dispensing animals. It has been revealed that the occurrence of fruit color is largely independent of phylogeny; instead, it is shaped by abiotic factors and the interaction with seed-dispersers (Valenta et al. 2018). Moreover, the emission of carotenoid cleavage products (such as β-ionone and 6-methyl-5-hepten-2-one) together with other volatiles suggests the ripening of fruits and also attracts seed-dispersing organisms (Dudareva et al. 2006; Goff and Klee 2006)

### Carotenoids derived classic hormone: ABA

Abscisic acid (ABA) is a carotenoid derived hormone that has been extensively studied and regulates many physiological activities in plants including stomata movement, seed germination, root development, and leaf senescence as well as responses to abiotic and biotic stresses (Finkelstein 2013; Chen et al. 2020) (Fig. 4). ABA can be detected in several cyanobacteria and algae. However, only in green algae, the common substrate 9’-*cis*-neoxanthin for ABA biosynthesis in higher plants can be detected (Giossi et al. 2020).

ABA is perceived by a family of PYR/PYL receptors (Park et al. 2009; Cutler et al. 2010). Most algae genomes do not encode PYL-like proteins except a few. Those PYL-like proteins present PP2C activities but are independent from ABA, which may represent the ancestral function of PYL (de Vries et al. 2018; Cheng et al. 2019). Meanwhile, a ligand-independent origin of abscisic acid perception by ABA-mediated fine-tuning of the PP2C–SnRK2 signaling cascade through PYL is a key evolutionary hallmark for land plants to conquer variable stresses (Blázquez et al. 2020).

### Carotenoid derived new hormone: strigolactones

Strigolactones (SLs) are another class of carotenoid-derived plant hormones initially found promoting symbiotic interactions with arbuscular mycorrhizal fungi (Akiyama et al. 2005). As a new class of phytohormones, SLs regulate axillary shoot growth, leaf senescence, and root architecture among many functions in land plants (Gomez-Roldan et al. 2008; Al-Babili and Bouwmeester 2015; Bürger and Chory 2020; Moreno et al. 2021) (Fig. 4). The synthesis of SLs from carotenoids in plants primarily involves a core pathway including a carotene isomerase (DWARF27), CCD7, CCD8 and a cytochrome P450 enzyme (MAX1) (Alder et al. 2012; Abe et al. 2014; Abuauf et al. 2018). In some groups of charophyte algae and *Physcomitrella patens*, SLs can also be identified but no CCD8 orthologues have been found (Proust et al. 2011; Delaux et al. 2012), suggesting alternative pathways for SL biosynthesis in the evolutionary ancestors of plants (Waters et al. 2017).

Intensive efforts have contributed to the mechanistic understanding of how SL works through receptors to trigger downstream response (Waters et al. 2017; Moreno et al. 2021). The findings of physical interaction between transcriptional repressor D53 and SL receptor D14 connect the signal perceiving and gene expression (Jiang et al. 2013; Zhou et al. 2013). As the receptor of SLs, D14 has relatively relaxed structural requirements and is possibly the result of gradual neo-functionalization within the D14-like protein family (Waters et al. 2017). D14 has both hydrolase activity to cleave SLs and SL perception ability, which is uncommon for phytohormone receptors. A recent study describes the sequential action of the D14 dual functions, which D14 deactivates bioactive SLs by the hydrolytic cleavage after signal perception (Seto et al. 2019).

### Apocarotenoid signaling: how does it work?

Plants have recruited apocarotenoids as signals or regulators during evolution (Wang et al. 2021a). In addition to ABA and SLs, an array of known and unidentified apocarotenoids act as signaling molecules or regulators to affect plant growth and development and in response to environmental stresses, although the roles of vast apocarotenoids remain unknown (Hou et al. 2016; D'Alessandro and Havaux 2019; Havaux 2020; Moreno et al. 2021) (Fig. 4). β-cyclocitral, a volatile apocarotenoid derived from either non-enzymatic or enzymatic oxidation of β-carotene, serve as a signal in response to abiotic stresses (Ramel et al. 2012; Shumbe et al. 2014). It is also a conserved root growth regulator (Dickinson et al. 2019) and enhances resistance to herbivores along inhibition of DXS activity by binding to its cofactor binding site (Mitra et al. 2021). Zaxinone cleaved from zeaxanthin is a novel growth regulator distributed in many plants (Mi et al. 2018; Wang et al. 2019; Ablazov et al. 2020). Anchorene derived from β-carotene is a new signaling molecule required for anchor root development by modulating the auxin distribution (Jia et al. 2019a; Jia et al. 2021). Besides, there are some *cis*-carotene-derived unidentified apocarotenoid signals to mediate leaf and plastid development in plants (Avendano-Vazquez et al. 2014; Alagoz et al. 2018; Cazzonelli et al. 2020; Escobar-Tovar et al. 2021; Moreno et al. 2021).

One key question to fully understand apocarotenoid signaling is: how do cells perceive those signals? It is possible that more apocarotenoid receptors may exist following successful isolation of strigolactone and ABA receptors. In mammalian cells, it has been demonstrated that retinoid receptors can bind β-apocarotenoids (Eroglu 2012; Harrison and Quadro 2018). Indeed, simply mutation of three amino acids can repurpose a plant karrikin receptor to a strigolactone receptor (Arellano-Saab et al. 2021), which imply the flexibility of apocarotenoid receptors. Whether carotenoid-derived hormone receptors can also recognize other apocarotenoid molecules need to be further studied. With the availability of high accuracy protein structures (Jumper et al. 2021), molecular docking of apocarotenoid signaling molecules to potential receptor proteins may provide some clues.

One way of apocarotenoid signaling is feedback regulation of the carotenoid metabolic pathway (Kachanovsky et al. 2012), although the mechanisms remain unknown. RNA structure can serve as a switch of protein translation in a ligand binding manner (de Jesus et al. 2021). The relatively small size and high mobility make apocarotenoids good candidates of RNA binding ligands to directly regulate protein expression. Whether this kind of apocarotenoid-dependent RNA switch is a universal mechanism needs to be further investigated.

## Horticultural and Agricultural application

Since carotenoids are important nutrients and phytonutrients, significant efforts have been made to generate crops enriched with carotenoids (Giuliano 2017; Zheng et al. 2020). The advances and innovations of approaches for metabolic engineering will facilitate production of more nutritious crops (Wurtzel 2019; Irfan et al. 2021). Crops in which the carotenoid pathway is modified also have the potential to be useful for horticultural and agricultural applications in developing new and/or improved varieties. Carotenoids provide plant organs with color. Alteration of carotenoid level and composition can generate crops with new color, expanding crop diversity. Since carotenoids are the precursors for the biosynthesis of phytohormones ABA and strigolactones, manipulation of the pathway genes can generate plants tolerant to stresses and/or change plant architectures. Examples include CRISPR/Cas9-mediated mutation of *CCD8* in tomato to develop host resistance to Broomrapes, a group of plant parasites that cause severe damage to crops (Bari et al. 2019), and overexpression of *LCYB2* to improve abiotic stress tolerance in sweetpotato (Kang et al. 2018). In addition, *CCD7* and *Z-ISO* mutants in rice have been shown to improve tiller number and grain yield (Liu et al. 2013; Zhou et al. 2021) and knockout of *CCD8* changes shoot architecture in grapevine (Ren et al. 2020).

Manipulation of pathway and regulatory genes may also improve some other desirable horticultural and agronomic traits. Heterologous expression of *GGPPS* enables fast plant growth, early flowering, and higher seed yield in plants (Tata et al. 2016). The *OR* gene as a major posttranslational regulator of PSY for carotenoid biosynthesis is a chaperone protein (Zhou et al. 2015a; Park et al. 2016; Chayut et al. 2017; Welsch et al. 2018). Its overexpression promotes early flowering, fruit set, and seed production in transgenic tomato (Yazdani et al. 2019) as well as enhances heat stress tolerance in sweetpotato plants (Kim et al. 2021). Expression of *LCYB* was also found to promote faster plant growth, early flowering, and increased biomass in tobacco (Moreno et al. 2020) and extended shelf life of tomato fruit (Diretto et al. 2020). These works suggest a substantial potential of genetic manipulation of carotenoid metabolic pathway and regulatory genes for horticultural and agricultural applications.

## Conclusion and key areas for future research

Significant progress has been achieved in our understanding of carotenoid metabolism and regulation as well as in elucidating the new functions of carotenoid metabolites in plant growth and development. The rapid technique advances also allow more crop systems to be taken advantage of to reveal the common and unique regulatory mechanisms. These bring carotenoid research to a golden era. Nevertheless, there are still various outstanding questions for future carotenoid research.

The identification and verification of intrinsic regulators of carotenoid metabolism are still a focusing area of carotenoid research. While some transcription factors and regulators have been shown to regulate the expression of carotenoid metabolic pathway genes or enzymes, gaps remain whether they are *bona fide* regulators, whether they function across plant species, or what their modes of action are in modulating carotenoid metabolism.

Both environmental and developmental signals regulate carotenogenic gene expression to affect carotenoid metabolism. Much remains unknown for what are the signaling pathways or how these signals control carotenogenesis.

Carotenogenic enzymes are believed to form enzyme complexes for efficiently driving the metabolic flux through the pathway. However, solid evidence for the enzyme complexes is lacking. Revealing carotenoid biosynthetic enzyme complexes and understanding their assembly will also be highly demanded in future.

Since carotenoids are essential photosynthetic pigments, synthesis of carotenoids in green tissue of plants must be under tight control with chlorophyll synthesis for optimal photosynthesis and chloroplast development. How these two biosynthesis pathways are coordinately regulated or what the common regulators are needs to be investigated.

The recent discovery of carotenoid derived apocarotenoid signals expands the current knowledge of plant signaling. Some basic questions need to be answered including: What are the identities of many unknown apocarotenoid signaling molecules? What are the perception mechanisms of the apocarotenoid signaling? How do the developmental and/or environmental cues trigger apocarotenoid signaling?

Since chromoplasts are the organelle for carotenoid accumulation, chromoplast development is critically important for high levels of carotenoid accumulation in many horticultural plants. Although the gain-of-function alleles of *OR* is known to trigger chromoplast formation, the nature of chromoplast biogenesis remains unknown, which needs to be further explored not only for plastid biology but also carotenoid enrichment in major food crops.

Carotenoids are nutritionally essential and health important. Biofortification of crops with carotenoids remains a main focus in carotenoid research. Better understanding of regulatory mechanisms and enzyme kinetics of carotenoid metabolism and chromoplast formation will steer both conventional breeding and genetic engineering of carotenoids. Knowledge of enzyme kinetics will guide the directed-evolution of more powerful carotenogenic enzymes, while the rapid-evolving gene editing technology will make the metabolic pathway optimization and redesign possible in crops. Since the biosynthesis, degradation and stable accumulation in plastids together define the carotenoid levels in plant organs, multi-target engineering will provide us more effective and precise ways for carotenoid biofortification in major crops. With more genetic tools and strategies added in the toolbox, more golden crops can be developed for better human nutrition and health. In addition, crops can be improved with some desirable horticultural and agronomic traits to enhance resilient agricultural system.

## Data Availability

Not applicable.

## Abbreviations

ABA: Abscisic acid
ARC3: ACCUMULATION AND REPLICATION OF CHLOROPLAST 3
BBX: B-box transcription factors
bHLH: basic Helix-Loop-Helix transcription factors
CCD: carotenoid cleavage dioxygenase
CCO: carotenoid cleavage oxygenase
CCS: capsanthin/capsorubin synthase
Clp: Clp protease
CRISPR: Clustered Regularly Interspaced short Palindromic Repeats
CRTISO: carotenoid isomerase
DMAPP: dimethylallyl pyrophosphate
DXS: 1-deoxy-D-xylulose 5-phosphate synthase
GGPP: geranylgeranyl diphosphate
HY5: ELONGATED HYPOCOTYL 5
IPP: isopentenyl pyrophosphate
JMJ6: Jumonji C-terminal (JmjC) domain-containing demethylase 6
LCYB: lycopene β‐cyclase
LCYE: lycopene ε‐cyclase
LHC: light-harvesting complexes
LHP1: LIKE HETEROCHROMATIN PROTEIN1
MADS: MADSbox containing transcription factors
MAX1: MORE AXILLARY GROWTH 1
MYB: Myeloblastosis family of transcription factors
NAC: NAM, ATAF, and CUC transcription factors
NCED: 9-cis-epoxycarotenoid dioxygenases
NOR: NON-RIPENING transcription factor
NPQ: non-photochemical quenching
NXS: neoxanthin synthase
OR: ORANGE protein
PDS: phytoene desaturase
PDV1: PLASTID DIVISION 1
PHL3: PHOSPHATE STARVATION RESPONSE FACTOR LIKE3
PIFs: PHYTOCHROME-INTERACTING FACTORS
PLB: prolamellar body
PSY: phytoene synthase
PP2C: PROTEIN PHOSPHATASE 2C
PPSR1: Plastid Protein Sensing RING E3 ligase 1
PTM: posttranslational modification
PYL: PYR1-LIKE
PYR: PYRABACTIN RESISTANCE
RAP2.2: APETALA2/ethylene response factor-type transcription factor 2.2
RCP1&2: REDUCED CAROTENOID PIGMENTATION 1&2
RIN: RIPENING INHIBITOR
SGR: STAY GREEN
SDG8: SET DOMAIN GROUP 8
SBP1: SQUAMOSA PROMOTER BINDING PROTEIN1
SL: strigolactone
TAGL1: TOMATO AGAMOUS-LIKE 1
VDE: violaxanthin de-epoxidase
WP1: WHITE PETAL1
WRKY: WRKYGQK motif-containing transcription factors
ZAS: zaxinone synthase
ZDS: ζ-carotene desaturase
ZISO: ζ-carotene isomerase

## References

[CR1] Abe S, Sado A, Tanaka K, Kisugi T, Asami K, Ota S, Kim HI, Yoneyama K, Xie X, Ohnishi T, Seto Y, Yamaguchi S, Akiyama K, Yoneyama K, Nomura T (2014). Carlactone is converted to carlactonoic acid by MAX1 in Arabidopsis and its methyl ester can directly interact with AtD14 in vitro. Proc Natl Acad Sci U S A.

[CR2] Ablazov A, Mi J, Jamil M, Jia KP, Wang JY, Feng Q, Al-Babili S (2020). The Apocarotenoid Zaxinone Is a Positive Regulator of Strigolactone and Abscisic Acid Biosynthesis in Arabidopsis Roots. Front Plant Sci.

[CR3] Abuauf H, Haider I, Jia K-P, Ablazov A, Mi J, Blilou I, Al-Babili S (2018). The Arabidopsis DWARF27 gene encodes an all-trans-/9-cis-β-carotene isomerase and is induced by auxin, abscisic acid and phosphate deficiency. Plant Sci.

[CR4] Ahn TK, Avenson TJ, Ballottari M, Cheng YC, Niyogi KK, Bassi R, Fleming GR (2008). Architecture of a charge-transfer state regulating light harvesting in a plant antenna protein. Science.

[CR5] Ahrazem O, Gomez-Gomez L, Rodrigo MJ, Avalos J, Limon MC (2016). Carotenoid Cleavage Oxygenases from Microbes and Photosynthetic Organisms: Features and Functions. Int J Mol Sci.

[CR6] Akiyama K, Matsuzaki K, Hayashi H (2005). Plant sesquiterpenes induce hyphal branching in arbuscular mycorrhizal fungi. Nature.

[CR7] Alagoz Y, Nayak P, Dhami N, Cazzonelli CI (2018). cis-carotene biosynthesis, evolution and regulation in plants: The emergence of novel signaling metabolites. Arch Biochem Biophys.

[CR8] Al-Babili S, Beyer P (2005). Golden Rice--five years on the road--five years to go?. Trends Plant Sci.

[CR9] Al-Babili S, Bouwmeester HJ (2015). Strigolactones, a novel carotenoid-derived plant hormone. Annu Rev Plant Biol.

[CR10] Alder A, Jamil M, Marzorati M, Bruno M, Vermathen M, Bigler P, Ghisla S, Bouwmeester H, Beyer P, Al-Babili S (2012). The path from beta-carotene to carlactone, a strigolactone-like plant hormone. Science.

[CR11] Álvarez D, Voß B, Maass D, Wüst F, Schaub P, Beyer P, Welsch R. Carotenogenesis Is Regulated by 5'UTR-Mediated Translation of Phytoene Synthase Splice Variants. Plant Physiol. 2016;172(4):2314-2326. 10.1104/pp.16.01262 10.1104/pp.16.01262 PMC5129717 27729470

[CR12] Ampomah-Dwamena C, Thrimawithana AH, Dejnoprat S, Lewis D, Espley RV, Allan AC (2019). A kiwifruit (Actinidia deliciosa) R2R3-MYB transcription factor modulates chlorophyll and carotenoid accumulation. New Phytol.

[CR13] Anwar S, Brenya E, Alagoz Y, Cazzonelli CI (2021). Epigenetic control of carotenogenesis during plant development. Crit Rev Plant Sci.

[CR14] Arango J, Jourdan M, Geoffriau E, Beyer P, Welsch R (2014). Carotene hydroxylase activity determines the levels of both α-carotene and total carotenoids in orange carrots. Plant Cell.

[CR15] Arellano-Saab A, Bunsick M, Al Galib H, Zhao W, Schuetz S, Bradley JM, et al. Three mutations repurpose a plant karrikin receptor to a strigolactone receptor. Proc Natl Acad Sci U S A. 2021;118. 10.1073/pnas.2103175118. 10.1073/pnas.2103175118 PMC8325247 34301902

[CR16] Auldridge ME, Block A, Vogel JT, Dabney-Smith C, Mila I, Bouzayen M, Magallanes-Lundback M, DellaPenna D, McCarty DR, Klee HJ (2006). Characterization of three members of the Arabidopsis carotenoid cleavage dioxygenase family demonstrates the divergent roles of this multifunctional enzyme family. Plant J.

[CR17] Avendano-Vazquez AO, Cordoba E, Llamas E, San Roman C, Nisar N, De la Torre S, Ramos-Vega M, Gutierrez-Nava MD, Cazzonelli CI, Pogson BJ, Leon P (2014). An Uncharacterized Apocarotenoid-Derived Signal Generated in zeta-Carotene Desaturase Mutants Regulates Leaf Development and the Expression of Chloroplast and Nuclear Genes in Arabidopsis. Plant Cell.

[CR18] Bai C, Capell T, Berman J, Medina V, Sandmann G, Christou P, Zhu C (2016). Bottlenecks in carotenoid biosynthesis and accumulation in rice endosperm are influenced by the precursor-product balance. Plant Biotechnol J.

[CR19] Bari VK, Nassar JA, Kheredin SM, Gal-On A, Ron M, Britt A, Steele D, Yoder J, Aly R (2019). CRISPR/Cas9-mediated mutagenesis of CAROTENOID CLEAVAGE DIOXYGENASE 8 in tomato provides resistance against the parasitic weed Phelipanche aegyptiaca. Sci Rep.

[CR20] Barja MV, Ezquerro M, Beretta S, Diretto G, Florez-Sarasa I, Feixes E, et al. Several geranylgeranyl diphosphate synthase isoforms supply metabolic substrates for carotenoid biosynthesis in tomato. New Phytol. 2021. 10.1111/nph.17283. 10.1111/nph.17283 33590894

[CR21] Barja MV, Rodriguez-Concepcion M. Plant geranylgeranyl diphosphate synthases: every (gene) family has a story. aBIOTECH. 2021:1–10. 10.1007/s42994-021-00050-5. 10.1007/s42994-021-00050-5 PMC9590577 36303884

[CR22] Baseggio M, Murray M, Magallanes-Lundback M, Kaczmar N, Chamness J, Buckler ES, Smith ME, DellaPenna D, Tracy WF, Gore MA (2020). Natural variation for carotenoids in fresh kernels is controlled by uncommon variants in sweet corn. Plant Genome.

[CR23] Beisel KG, Jahnke S, Hofmann D, Koppchen S, Schurr U, Matsubara S (2010). Continuous turnover of carotenes and chlorophyll a in mature leaves of Arabidopsis revealed by 14CO2 pulse-chase labeling. Plant Physiol.

[CR24] Beltran JC, Stange C (2016). Apocarotenoids: A New Carotenoid-Derived Pathway. Subcell Biochem.

[CR25] Berry HM, Rickett DV, Baxter CJ, Enfissi EMA, Fraser PD (2019). Carotenoid biosynthesis and sequestration in red chilli pepper fruit and its impact on colour intensity traits. J Exp Bot.

[CR26] Blázquez MA, Nelson DC, Weijers D (2020). Evolution of plant hormone response pathways. Annu Rev Plant Biol.

[CR27] Bou-Torrent J, Toledo-Ortiz G, Ortiz-Alcaide M, Cifuentes-Esquivel N, Halliday KJ, Martinez-Garcia JF, Rodriguez-Concepcion M (2015). Regulation of Carotenoid Biosynthesis by Shade Relies on Specific Subsets of Antagonistic Transcription Factors and Cofactors. Plant Physiol.

[CR28] Breitenbach J, Gerjets T, Sandmann G (2013). Catalytic properties and reaction mechanism of the CrtO carotenoid ketolase from the cyanobacterium Synechocystis sp. PCC 6803. Arch Biochem Biophys.

[CR29] Bürger M, Chory J (2020). The many models of strigolactone signaling. Trends Plant Sci.

[CR30] Bykowski M, Mazur R, Wojtowicz J, Suski S, Garstka M, Mostowska A, Kowalewska L (2021). Too rigid to fold: Carotenoid-dependent decrease in thylakoid fluidity hampers the formation of chloroplast grana. Plant Physiol.

[CR31] Camagna M, Grundmann A, Bar C, Koschmieder J, Beyer P, Welsch R (2019). Enzyme Fusion Removes Competition for Geranylgeranyl Diphosphate in Carotenogenesis. Plant Physiol.

[CR32] Cao H, Luo H, Yuan H, Eissa MA, Thannhauser TW, Welsch R, Hao YJ, Cheng L, Li L (2019). A Neighboring Aromatic-Aromatic Amino Acid Combination Governs Activity Divergence between Tomato Phytoene Synthases. Plant Physiol.

[CR33] Cardona T, Sanchez-Baracaldo P, Rutherford AW, Larkum AW (2019). Early Archean origin of Photosystem II. Geobiology.

[CR34] Cazzonelli CI, Cuttriss AJ, Cossetto SB, Pye W, Crisp P, Whelan J, Finnegan EJ, Turnbull C, Pogson BJ (2009). Regulation of carotenoid composition and shoot branching in Arabidopsis by a chromatin modifying histone methyltransferase, SDG8. Plant Cell.

[CR35] Cazzonelli CI, Hou X, Alagoz Y, Rivers J, Dhami N, Lee J, Marri S, Pogson BJ (2020). A cis-carotene derived apocarotenoid regulates etioplast and chloroplast development. Elife.

[CR36] Cazzonelli CI, Pogson BJ (2010). Source to sink: regulation of carotenoid biosynthesis in plants. Trends Plant Sci.

[CR37] Chayut N, Yuan H, Ohali S, Meir A, Sa'ar U, Tzuri G, Zheng Y, Mazourek M, Gepstein S, Zhou X, Portnoy V, Lewinsohn E, Schaffer AA, Katzir N, Fei Z, Welsch R, Li L, Burger J, Tadmor Y (2017). Distinct Mechanisms of the ORANGE Protein in Controlling Carotenoid Flux. Plant Physiol.

[CR38] Chayut N, Yuan H, Ohali S, Meir A, Yeselson Y, Portnoy V, Zheng Y, Fei Z, Lewinsohn E, Katzir N, Schaffer AA, Gepstein S, Burger J, Li L, Tadmor Y (2015). A bulk segregant transcriptome analysis reveals metabolic and cellular processes associated with Orange allelic variation and fruit beta-carotene accumulation in melon fruit. BMC Plant Biol.

[CR39] Chayut N, Yuan H, Saar Y, Zheng Y, Sun T, Zhou X, Hermanns A, Oren E, Faigenboim A, Hui M, Fei Z, Mazourek M, Burger J, Tadmor Y, Li L (2021). Comparative transcriptome analyses shed light on carotenoid production and plastid development in melon fruit. Hortic Res.

[CR40] Chen K, Li GJ, Bressan RA, Song CP, Zhu JK, Zhao Y (2020). Abscisic acid dynamics, signaling, and functions in plants. J Integr Plant Biol.

[CR41] Chen WC, Wang Q, Cao TJ, Lu S (2021). UBC19 is a new interacting protein of ORANGE for its nuclear localization in Arabidopsis thaliana. Plant Signal Behav.

[CR42] Cheng S, Xian W, Fu Y, Marin B, Keller J, Wu T, Sun W, Li X, Xu Y, Zhang Y, Wittek S, Reder T, Gunther G, Gontcharov A, Wang S, Li L, Liu X, Wang J, Yang H, Xu X, Delaux PM, Melkonian B, Wong GK, Melkonian M (2019). Genomes of Subaerial Zygnematophyceae Provide Insights into Land Plant Evolution. Cell.

[CR43] Coe KM, Ellison S, Senalik D, Dawson J, Simon P (2021). The influence of the Or and Carotene Hydroxylase genes on carotenoid accumulation in orange carrots [Daucus carota (L.)]. Theor Appl Genet.

[CR44] Collini E (2019). Carotenoids in Photosynthesis: The Revenge of the “Accessory” Pigments. Chem.

[CR45] Cutler SR, Rodriguez PL, Finkelstein RR, Abrams SR (2010). Abscisic acid: emergence of a core signaling network. Annu Rev Plant Biol.

[CR46] D'Alessandro S, Havaux M (2019). Sensing beta-carotene oxidation in photosystem II to master plant stress tolerance. New Phytol.

[CR47] D'Alessandro S, Ksas B, Havaux M (2018). Decoding beta-Cyclocitral-Mediated Retrograde Signaling Reveals the Role of a Detoxification Response in Plant Tolerance to Photooxidative Stress. Plant Cell.

[CR48] D'Andrea L, Simon-Moya M, Llorente B, Llamas E, Marro M, Loza-Alvarez P, Li L, Rodriguez-Concepcion M (2018). Interference with Clp protease impairs carotenoid accumulation during tomato fruit ripening. J Exp Bot.

[CR49] de Jesus V, Qureshi NS, Warhaut S, Bains JK, Dietz MS, Heilemann M, Schwalbe H, Fürtig B (2021). Switching at the ribosome: riboswitches need rProteins as modulators to regulate translation. Nat Commun.

[CR50] de Vries J, Curtis BA, Gould SB, Archibald JM (2018). Embryophyte stress signaling evolved in the algal progenitors of land plants. Proc Natl Acad Sci U S A.

[CR51] Delaux PM, Xie X, Timme RE, Puech-Pages V, Dunand C, Lecompte E, Delwiche CF, Yoneyama K, Becard G, Sejalon-Delmas N (2012). Origin of strigolactones in the green lineage. New Phytol.

[CR52] Demurtas OC, de Brito FR, Diretto G, Ferrante P, Frusciante S, Pietrella M, Aprea G, Borghi L, Feeney M, Frigerio L, Coricello A, Costa G, Alcaro S, Martinoia E, Giuliano G (2019). ABCC Transporters Mediate the Vacuolar Accumulation of Crocins in Saffron Stigmas. Plant Cell.

[CR53] Dhami N, Cazzonelli CI. Environmental impacts on carotenoid metabolism in leaves. Plant Growth Regul. 2020:1–23. 10.1007/s10725-020-00661-w.

[CR54] Dhar MK, Mishra S, Bhat A, Chib S, Kaul S (2020). Plant carotenoid cleavage oxygenases: structure-function relationships and role in development and metabolism. Brief Funct Genomics.

[CR55] Dickinson AJ, Lehner K, Mi J, Jia K-P, Mijar M, Dinneny J, Al-Babili S, Benfey PN (2019). β-Cyclocitral is a conserved root growth regulator. Proc Natl Acad Sci U S A.

[CR56] Diretto G, Ahrazem O, Rubio-Moraga A, Fiore A, Sevi F, Argandona J, Gomez-Gomez L (2019). UGT709G1: a novel uridine diphosphate glycosyltransferase involved in the biosynthesis of picrocrocin, the precursor of safranal in saffron (Crocus sativus). New Phytol.

[CR57] Diretto G, Frusciante S, Fabbri C, Schauer N, Busta L, Wang Z, Matas AJ, Fiore A, KC Rose J, Fernie AR. (2020). Manipulation of β-carotene levels in tomato fruits results in increased ABA content and extended shelf life. Plant Biotechnol J.

[CR58] Diretto G, Welsch R, Tavazza R, Mourgues F, Pizzichini D, Beyer P, Giuliano G (2007). Silencing of beta-carotene hydroxylase increases total carotenoid and beta-carotene levels in potato tubers. BMC Plant Biol.

[CR59] Dudareva N, Negre F, Nagegowda DA, Orlova I (2006). Plant volatiles: recent advances and future perspectives. Crit Rev Plant Sci.

[CR60] Durek P, Schmidt R, Heazlewood JL, Jones A, MacLean D, Nagel A, Kersten B, Schulze WX (2010). PhosPhAt: the Arabidopsis thaliana phosphorylation site database. An update. Nucleic Acids Res.

[CR61] Dutta S, Muthusamy V, Chhabra R, Baveja A, Zunjare RU, Mondal TK, Yadava DK, Hossain F (2021). Low expression of carotenoids cleavage dioxygenase 1 (ccd1) gene improves the retention of provitamin-A in maize grains during storage. Mol Gen Genomics.

[CR62] Edwards MB, Choi GP, Derieg NJ, Min Y, Diana AC, Hodges SA, et al. Genetic architecture of floral traits in bee-and hummingbird-pollinated sister species of Aquilegia (columbine). bioRxiv. 2021. 10.1101/2021.04.12.439277. 10.1111/evo.14313 34270789

[CR63] Egea I, Barsan C, Bian W, Purgatto E, Latche A, Chervin C, Bouzayen M, Pech JC (2010). Chromoplast differentiation: current status and perspectives. Plant Cell Physiol.

[CR64] Egea I, Bian W, Barsan C, Jauneau A, Pech JC, Latche A, Li Z, Chervin C (2011). Chloroplast to chromoplast transition in tomato fruit: spectral confocal microscopy analyses of carotenoids and chlorophylls in isolated plastids and time-lapse recording on intact live tissue. Ann Bot.

[CR65] Eggersdorfer M, Wyss A (2018). Carotenoids in human nutrition and health. Arch Biochem Biophys.

[CR66] Ellison S, Luby C, Corak K, Coe K, Senalik D, Iorizzo M, Goldman I, Simon P, Dawson J (2018). Association analysis reveals the importance of the Or gene in carrot (Daucus carota L.) carotenoid presence and domestication. Genetics.

[CR67] Enfissi EM, Nogueira M, Bramley PM, Fraser PD (2017). The regulation of carotenoid formation in tomato fruit. Plant J.

[CR68] Eroglu A (2012). Apocarotenoids modulate retinoid receptors: The Ohio State University.

[CR69] Escobar-Tovar L, Sierra J, Hernandez-Munoz A, McQuinn RP, Mathioni S, Cordoba E, Colas des Francs-Small C, Meyers BC, Pogson B, Leon P (2021). Deconvoluting apocarotenoid-mediated retrograde signaling networks regulating plastid translation and leaf development. Plant J.

[CR70] Estevez JM, Cantero A, Reindl A, Reichler S, Leon P (2001). 1-Deoxy-D-xylulose-5-phosphate synthase, a limiting enzyme for plastidic isoprenoid biosynthesis in plants. J Biol Chem.

[CR71] Falchi R, Vendramin E, Zanon L, Scalabrin S, Cipriani G, Verde I, Vizzotto G, Morgante M (2013). Three distinct mutational mechanisms acting on a single gene underpin the origin of yellow flesh in peach. Plant J.

[CR72] Fantini E, Falcone G, Frusciante S, Giliberto L, Giuliano G (2013). Dissection of tomato lycopene biosynthesis through virus-induced gene silencing. Plant Physiol.

[CR73] Feder A, Chayut N, Gur A, Freiman Z, Tzuri G, Meir A, Saar U, Ohali S, Baumkoler F, Gal-On A (2019). The role of carotenogenic metabolic flux in carotenoid accumulation and chromoplast differentiation: lessons from the melon fruit. Front Plant Sci.

[CR74] Felemban A, Braguy J, Zurbriggen MD, Al-Babili S (2019). Apocarotenoids Involved in Plant Development and Stress Response. Front Plant Sci.

[CR75] Finkelstein R (2013). Abscisic Acid synthesis and response. Arabidopsis Book.

[CR76] Fiorilli V, Wang JY, Bonfante P, Lanfranco L, Al-Babili S (2019). Apocarotenoids: Old and New Mediators of the Arbuscular Mycorrhizal Symbiosis. Front Plant Sci.

[CR77] Floris D, Kuhlbrandt W (2021). Molecular landscape of etioplast inner membranes in higher plants. Nat Plants.

[CR78] Frachon L, Stirling SA, Schiestl FP, Dudareva N (2021). Combining biotechnology and evolution for understanding the mechanisms of pollinator attraction. Curr Opin Biotechnol.

[CR79] Frank HA, Cogdell RJ (1996). Carotenoids in photosynthesis. Photochem Photobiol.

[CR80] Fraser PD, Schuch W, Bramley PM (2000). Phytoene synthase from tomato (Lycopersicon esculentum) chloroplasts--partial purification and biochemical properties. Planta.

[CR81] Fray RG, Grierson D (1993). Identification and genetic analysis of normal and mutant phytoene synthase genes of tomato by sequencing, complementation and co-suppression. Plant Mol Biol.

[CR82] Frusciante S, Diretto G, Bruno M, Ferrante P, Pietrella M, Prado-Cabrero A, Rubio-Moraga A, Beyer P, Gomez-Gomez L, Al-Babili S, Giuliano G (2014). Novel carotenoid cleavage dioxygenase catalyzes the first dedicated step in saffron crocin biosynthesis. Proc Natl Acad Sci U S A.

[CR83] Fujiwara Y, Hashimoto K, Manabe K, Maoka T (2002). Structures of tobiraxanthins A1, A2, A3, B, C and D, new carotenoids from the seeds of Pittosporum tobira. Tetrahedron Lett.

[CR84] Galpaz N, Burger Y, Lavee T, Tzuri G, Sherman A, Melamed T, Eshed R, Meir A, Portnoy V, Bar E, Shimoni-Shor E, Feder A, Saar Y, Saar U, Baumkoler F, Lewinsohn E, Schaffer AA, Katzir N, Tadmor Y (2013). Genetic and chemical characterization of an EMS induced mutation in Cucumis melo CRTISO gene. Arch Biochem Biophys.

[CR85] Gao J, Yang S, Tang K, Li G, Gao X, Liu B, Wang S, Feng X (2021). GmCCD4 controls carotenoid content in soybeans. Plant Biotechnol J.

[CR86] Gao L, Gonda I, Sun H, Ma Q, Bao K, Tieman DM, Burzynski-Chang EA, Fish TL, Stromberg KA, Sacks GL, Thannhauser TW, Foolad MR, Diez MJ, Blanca J, Canizares J, Xu Y, van der Knaap E, Huang S, Klee HJ, Giovannoni JJ, Fei Z (2019). The tomato pan-genome uncovers new genes and a rare allele regulating fruit flavor. Nat Genet.

[CR87] García-Cerdán JG, Schmid EM, Takeuchi T, McRae I, McDonald KL, Yordduangjun N, Hassan AM, Grob P, Xu CS, Hess HF (2020). Chloroplast Sec14-like 1 (CPSFL1) is essential for normal chloroplast development and affects carotenoid accumulation in Chlamydomonas. Proc Natl Acad Sci U S A.

[CR88] Gemenet DC, da Silva Pereira G, De Boeck B, Wood JC, Mollinari M, Olukolu BA, Diaz F, Mosquera V, Ssali RT, David M, Kitavi MN, Burgos G, Felde TZ, Ghislain M, Carey E, Swanckaert J, LJM C, Fei Z, Hamilton JP, Yada B, Yencho GC, Zeng ZB, ROM M, Khan A, Gruneberg WJ, Buell CR (2020). Quantitative trait loci and differential gene expression analyses reveal the genetic basis for negatively associated beta-carotene and starch content in hexaploid sweetpotato [Ipomoea batatas (L.) Lam.]. Theor Appl Genet.

[CR89] Ghosh S, Bishop MM, Roscioli JD, LaFountain AM, Frank HA, Beck WF (2017). Excitation Energy Transfer by Coherent and Incoherent Mechanisms in the Peridinin-Chlorophyll a Protein. J Phys Chem Lett.

[CR90] Giossi C, Cartaxana P, Cruz S (2020). Photoprotective role of neoxanthin in plants and algae. Molecules.

[CR91] Giuliano G (2017). Provitamin A biofortification of crop plants: a gold rush with many miners. Curr Opin Biotechnol.

[CR92] Goff SA, Klee HJ (2006). Plant volatile compounds: sensory cues for health and nutritional value?. Science.

[CR93] Gomez-Roldan V, Fermas S, Brewer PB, Puech-Pages V, Dun EA, Pillot JP, Letisse F, Matusova R, Danoun S, Portais JC, Bouwmeester H, Becard G, Beveridge CA, Rameau C, Rochange SF (2008). Strigolactone inhibition of shoot branching. Nature.

[CR94] Gong J, Zeng Y, Meng Q, Guan Y, Li C, Yang H, et al. Red light-induced kumquat fruit colouration is attributable to increased carotenoid metabolism regulated by FcrNAC22. J Exp Bot. 2021. 10.1093/jxb/erab283. 10.1093/jxb/erab283 34125891

[CR95] Gonzalez-Jorge S, Ha S-H, Magallanes-Lundback M, Gilliland LU, Zhou A, Lipka AE, Nguyen Y-N, Angelovici R, Lin H, Cepela J (2013). Carotenoid cleavage dioxygenase4 is a negative regulator of β-carotene content in Arabidopsis seeds. Plant Cell.

[CR96] Grabsztunowicz M, Koskela MM, Mulo P (2017). Post-translational modifications in regulation of chloroplast function: recent advances. Front Plant Sci.

[CR97] Harjes CE, Rocheford TR, Bai L, Brutnell TP, Kandianis CB, Sowinski SG, Stapleton AE, Vallabhaneni R, Williams M, Wurtzel ET, Yan J, Buckler ES (2008). Natural genetic variation in lycopene epsilon cyclase tapped for maize biofortification. Science.

[CR98] Harrison EH, Quadro L (2018). Apocarotenoids: Emerging Roles in Mammals. Annu Rev Nutr.

[CR99] Hashimoto H, Uragami C, Cogdell RJ (2016). Carotenoids and Photosynthesis. Subcell Biochem.

[CR100] Havaux M (1998). Carotenoids as membrane stabilizers in chloroplasts. Trends Plant Sci.

[CR101] Havaux M (2020). beta-Cyclocitral and derivatives: Emerging molecular signals serving multiple biological functions. Plant Physiol Biochem.

[CR102] Havaux M, Gruszecki WI (1993). Heat-and light-induced chlorophyll a fluorescence changes in potato leaves containing high or low levels of the carotenoid zeaxanthin: Indications of a regulatory effect of zeaxanthin on thylakoid membrane fluidity. Photochem Photobiol.

[CR103] Hemmerlin A (2013). Post-translational events and modifications regulating plant enzymes involved in isoprenoid precursor biosynthesis. Plant Sci.

[CR104] Hermanns AS, Zhou XS, Xu Q, Tadmor Y, Li L (2020). Carotenoid Pigment Accumulation in Horticultural Plants. Hortic Plant J.

[CR105] Holt NE, Zigmantas D, Valkunas L, Li XP, Niyogi KK, Fleming GR (2005). Carotenoid cation formation and the regulation of photosynthetic light harvesting. Science.

[CR106] Horner HT, Healy RA, Ren G, Fritz D, Klyne A, Seames C, Thornburg RW (2007). Amyloplast to chromoplast conversion in developing ornamental tobacco floral nectaries provides sugar for nectar and antioxidants for protection. Am J Bot.

[CR107] Hou X, Rivers J, Leon P, McQuinn RP, Pogson BJ (2016). Synthesis and Function of Apocarotenoid Signals in Plants. Trends Plant Sci.

[CR108] Irfan M, Chavez B, Rizzo P, D’Auria JC, Moghe GD. Evolution-aided engineering of plant specialized metabolism. aBIOTECH. 2021. 10.1007/s42994-021-00052-3. 10.1007/s42994-021-00052-3 PMC9590541 36303885

[CR109] Isaacson T, Ronen G, Zamir D, Hirschberg J (2002). Cloning of *tangerine* from tomato reveals a carotenoid isomerase essential for the production of b-carotene and xanthophylls in plants. Plant Cell.

[CR110] Jahns P, Holzwarth AR (2012). The role of the xanthophyll cycle and of lutein in photoprotection of photosystem II. Biochim Biophys Acta.

[CR111] Jia KP, Baz L, Al-Babili S (2018). From carotenoids to strigolactones. J Exp Bot.

[CR112] Jia K-P, Dickinson AJ, Mi J, Cui G, Xiao TT, Kharbatia NM, Guo X, Sugiono E, Aranda M, Blilou I (2019). Anchorene is a carotenoid-derived regulatory metabolite required for anchor root formation in Arabidopsis. Sci Adv.

[CR113] Jia K-P, Li C, Bouwmeester HJ, Al-Babili S. Strigolactone biosynthesis and signal transduction. Strigolactones-Biol Appl. 2019b:1–45. 10.1007/978-3-030-12153-2_1.

[CR114] Jia K-P, Mi J, Ablazov A, Ali S, Yang Y, Balakrishna A, Berqdar L, Feng Q, Blilou I, Al-Babili S (2021). Iso-anchorene is an endogenous metabolite that inhibits primary root growth in Arabidopsis. Plant J.

[CR115] Jiang L, Liu X, Xiong G, Liu H, Chen F, Wang L, Meng X, Liu G, Yu H, Yuan Y, Yi W, Zhao L, Ma H, He Y, Wu Z, Melcher K, Qian Q, Xu HE, Wang Y, Li J (2013). DWARF 53 acts as a repressor of strigolactone signalling in rice. Nature.

[CR116] Jumper J, Evans R, Pritzel A, Green T, Figurnov M, Ronneberger O, Tunyasuvunakool K, Bates R, Zidek A, Potapenko A, Bridgland A, Meyer C, SAA K, Ballard AJ, Cowie A, Romera-Paredes B, Nikolov S, Jain R, Adler J, Back T, Petersen S, Reiman D, Clancy E, Zielinski M, Steinegger M, Pacholska M, Berghammer T, Bodenstein S, Silver D, Vinyals O, Senior AW, Kavukcuoglu K, Kohli P, Hassabis D (2021). Highly accurate protein structure prediction with AlphaFold. Nature.

[CR117] Kachanovsky DE, Filler S, Isaacson T, Hirschberg J (2012). Epistasis in tomato color mutations involves regulation of phytoene synthase 1 expression by cis-carotenoids. Proc Natl Acad Sci U S A.

[CR118] Kang C, He S, Zhai H, Li R, Zhao N, Liu Q (2018). A sweetpotato auxin response factor gene (IbARF5) is involved in carotenoid biosynthesis and salt and drought tolerance in transgenic Arabidopsis. Front Plant Sci.

[CR119] Kay KM, Sargent RD (2009). The Role of Animal Pollination in Plant Speciation: Integrating Ecology, Geography, and Genetics. Annu Rev Ecol Evol Syst.

[CR120] Kim HS, Ji CY, Lee CJ, Kim SE, Park SC, Kwak SS (2018). Orange: a target gene for regulating carotenoid homeostasis and increasing plant tolerance to environmental stress in marginal lands. J Exp Bot.

[CR121] Kim SE, Lee CJ, Park SU, Lim YH, Park WS, Kim HJ, Ahn MJ, Kwak SS, Kim HS (2021). Overexpression of the Golden SNP-Carrying Orange Gene Enhances Carotenoid Accumulation and Heat Stress Tolerance in Sweetpotato Plants. Antioxidants (Basel).

[CR122] Koschmieder J, Wüst F, Schaub P, Álvarez D, Trautmann D, Krischke M, Rustenholz C, Ji M, Mueller MJ, Bartels D (2021). Plant apocarotenoid metabolism utilizes defense mechanisms against reactive carbonyl species and xenobiotics. Plant Physiol.

[CR123] Lang Z, Wang Y, Tang K, Tang D, Datsenka T, Cheng J, Zhang Y, Handa AK, Zhu JK (2017). Critical roles of DNA demethylation in the activation of ripening-induced genes and inhibition of ripening-repressed genes in tomato fruit. Proc Natl Acad Sci U S A.

[CR124] Lee SY, Jang SJ, Jeong HB, Lee SY, Venkatesh J, Lee JH, et al. A mutation in Zeaxanthin epoxidase contributes to orange coloration and alters carotenoid contents in pepper fruit (Capsicum annuum). Plant J. 2021. 10.1111/tpj.15264. 10.1111/tpj.15264 33825226

[CR125] Li L, Paolillo DJ, Parthasarathy MV, Dimuzio EM, Garvin DF (2001). A novel gene mutation that confers abnormal patterns of beta-carotene accumulation in cauliflower (*Brassica oleracea* var. *botrytis*). Plant J.

[CR126] Li L, Yang Y, Xu Q, Owsiany K, Welsch R, Chitchumroonchokchai C, Lu S, Van Eck J, Deng XX, Failla M, Thannhauser TW (2012). The Or gene enhances carotenoid accumulation and stability during post-harvest storage of potato tubers. Mol Plant.

[CR127] Li L, Yuan H (2013). Chromoplast biogenesis and carotenoid accumulation. Arch Biochem Biophys.

[CR128] Li L, Yuan H, Zeng Y, Xu Q. Plastids and Carotenoid Accumulation. Carotenoids Nat. 2016:273–93. Springer. 10.1007/978-3-319-39126-7_10. 10.1007/978-3-319-39126-7_10 27485226

[CR129] Li Y, Beisson F, Pollard M, Ohlrogge J (2006). Oil content of Arabidopsis seeds: the influence of seed anatomy, light and plant-to-plant variation. Phytochemistry.

[CR130] Li Z, Jiang G, Liu X, Ding X, Zhang D, Wang X, Zhou Y, Yan H, Li T, Wu K, Jiang Y, Duan X (2020). Histone demethylase SlJMJ6 promotes fruit ripening by removing H3K27 methylation of ripening-related genes in tomato. New Phytol.

[CR131] Liang MH, He YJ, Liu DM, Jiang JG (2021). Regulation of carotenoid degradation and production of apocarotenoids in natural and engineered organisms. Crit Rev Biotechnol.

[CR132] Liang MH, Zhu J, Jiang JG (2018). Carotenoids biosynthesis and cleavage related genes from bacteria to plants. Crit Rev Food Sci Nutr.

[CR133] Liang Q, Deng H, Li Y, Liu Z, Shu P, Fu R, Zhang Y, Pirrello J, Zhang Y, Grierson D, Bouzayen M, Liu Y, Liu M (2020). Like Heterochromatin Protein 1b represses fruit ripening via regulating the H3K27me3 levels in ripening-related genes in tomato. New Phytol.

[CR134] Ling Q, Sadali NM, Soufi Z, Zhou Y, Huang B, Zeng Y, Rodriguez-Concepcion M, Jarvis RP (2021). The chloroplast-associated protein degradation pathway controls chromoplast development and fruit ripening in tomato. Nat Plants.

[CR135] Liu G, Li C, Yu H, Tao P, Yuan L, Ye J, Chen W, Wang Y, Ge P, Zhang J, Zhou G, Zheng W, Ye Z, Zhang Y (2020). GREEN STRIPE, encoding methylated TOMATO AGAMOUS-LIKE 1, regulates chloroplast development and Chl synthesis in fruit. New Phytol.

[CR136] Liu J, Novero M, Charnikhova T, Ferrandino A, Schubert A, Ruyter-Spira C, Bonfante P, Lovisolo C, Bouwmeester HJ, Cardinale F (2013). Carotenoid cleavage dioxygenase 7 modulates plant growth, reproduction, senescence, and determinate nodulation in the model legume Lotus japonicus. J Exp Bot.

[CR137] Liu L, Shao Z, Zhang M, Wang Q (2015). Regulation of carotenoid metabolism in tomato. Mol Plant.

[CR138] Liu Z, Yan H, Wang K, Kuang T, Zhang J, Gui L, An X, Chang W (2004). Crystal structure of spinach major light-harvesting complex at 2.72 Å resolution. Nature.

[CR139] Llamas E, Pulido P, Rodriguez-Concepcion M (2017). Interference with plastome gene expression and Clp protease activity in Arabidopsis triggers a chloroplast unfolded protein response to restore protein homeostasis. PLoS Genet.

[CR140] Llorente B, Martinez-Garcia JF, Stange C, Rodriguez-Concepcion M (2017). Illuminating colors: regulation of carotenoid biosynthesis and accumulation by light. Curr Opin Plant Biol.

[CR141] Llorente B, Torres-Montilla S, Morelli L, Florez-Sarasa I, Matus JT, Ezquerro M, D'Andrea L, Houhou F, Majer E, Pico B, Cebolla J, Troncoso A, Fernie AR, Daros JA, Rodriguez-Concepcion M (2020). Synthetic conversion of leaf chloroplasts into carotenoid-rich plastids reveals mechanistic basis of natural chromoplast development. Proc Natl Acad Sci U S A.

[CR142] Lopez AB, Van Eck J, Conlin BJ, Paolillo DJ, O'Neill J, Li L (2008). Effect of the cauliflower *Or* transgene on carotenoid accumulation and chromoplast formation in transgenic potato tubers. J Exp Bot.

[CR143] Lopez AB, Yang Y, Thannhauser TW, Li L (2008). Phytoene desaturase is present in a large protein complex in the plastid membrane. Physiol Plant.

[CR144] López AJ, Frusciante S, Niza E, Ahrazem O, Rubio-Moraga Á, Diretto G, Gómez-Gómez L (2021). A New Glycosyltransferase Enzyme from Family 91, UGT91P3, Is Responsible for the Final Glucosylation Step of Crocins in Saffron (Crocus sativus L.). Int J Mol Sci.

[CR145] Lu S, Van Eck J, Zhou X, Lopex AB, O'Halloran DM, Cosman KM, Conlin B, Paolillo DJ, Garvin DF, Vrebalov J, Kochian LV, Kupper H, Earle ED, Cao J, Li L (2006). The cauliflower Or gene encodes a DnaJ cysteine-rich domain-containing protein that mediates high-levels of b-carotene accumulation. Plant Cell.

[CR146] Lu S, Ye J, Zhu K, Zhang Y, Zhang M, Xu Q, et al. A citrus phosphate starvation response factor CsPHL3 negatively regulates carotenoid metabolism. Plant Cell Physiol. 2021a. 10.1093/pcp/pcab007. 10.1093/pcp/pcab007 33493291

[CR147] Lu S, Ye J, Zhu K, Zhang Y, Zhang M, Xu Q, Deng X (2021). A fruit ripening-associated transcription factor CsMADS5 positively regulates carotenoid biosynthesis in citrus. J Exp Bot.

[CR148] Luan YT, Fu XM, Lu PJ, Grierson D, Xu CJ (2020). Molecular Mechanisms Determining the Differential Accumulation of Carotenoids in Plant Species and Varieties. Crit Rev Plant Sci.

[CR149] Luo Z, Zhang J, Li J, Yang C, Wang T, Ouyang B, Li H, Giovannoni J, Ye Z (2013). A STAY-GREEN protein SlSGR1 regulates lycopene and beta-carotene accumulation by interacting directly with SlPSY1 during ripening processes in tomato. New Phytol.

[CR150] Maass D, Arango J, Wast F, Beyer P, Welsch R (2009). Carotenoid crystal formation in Arabidopsis and carro troots caused by increased phytoene synthase protein levels. PLoS ONE.

[CR151] Martel C, Vrebalov J, Tafelmeyer P, Giovannoni JJ (2011). The tomato MADS-box transcription factor RIPENING INHIBITOR interacts with promoters involved in numerous ripening processes in a COLORLESS NONRIPENING-dependent manner. Plant Physiol.

[CR152] Meneghin E, Volpato A, Cupellini L, Bolzonello L, Jurinovich S, Mascoli V, Carbonera D, Mennucci B, Collini E (2018). Coherence in carotenoid-to-chlorophyll energy transfer. Nat Commun.

[CR153] Meng N, Wei Y, Gao Y, Yu K, Cheng J, Li XY, Duan CQ, Pan QH (2020). Characterization of Transcriptional Expression and Regulation of Carotenoid Cleavage Dioxygenase 4b in Grapes. Front Plant Sci.

[CR154] Meng Y, Wang Z, Wang Y, Wang C, Zhu B, Liu H, Ji W, Wen J, Chu C, Tadege M, Niu L, Lin H (2019). The MYB Activator WHITE PETAL1 Associates with MtTT8 and MtWD40-1 to Regulate Carotenoid-Derived Flower Pigmentation in Medicago truncatula. Plant Cell.

[CR155] Mi J, Jia KP, Wang JY, Al-Babili S (2018). A rapid LC-MS method for qualitative and quantitative profiling of plant apocarotenoids. Anal Chim Acta.

[CR156] Mitra S, Estrada-Tejedor R, Volke DC, Phillips MA, Gershenzon J, Wright LP. Negative regulation of plastidial isoprenoid pathway by herbivore-induced β-cyclocitral in Arabidopsis thaliana. Proc Natl Acad Sci U S A. 2021;118. 10.1073/pnas.2008747118. 10.1073/pnas.2008747118 PMC7958287 33674379

[CR157] Miyagishima SY (2020). A Multifunctional Modulator Coordinates Nuclear Transcription and Plastid Metabolism and Proliferation. Mol Plant.

[CR158] Moreno JC, Mi J, Agrawal S, Kossler S, Tureckova V, Tarkowska D, Thiele W, Al-Babili S, Bock R, Schottler MA (2020). Expression of a carotenogenic gene allows faster biomass production by redesigning plant architecture and improving photosynthetic efficiency in tobacco. Plant J.

[CR159] Moreno JC, Mi J, Alagoz Y, Al-Babili S (2021). Plant apocarotenoids: from retrograde signaling to interspecific communication. Plant J.

[CR160] Mortimer CL, Misawa N, Ducreux L, Campbell R, Bramley PM, Taylor M, Fraser PD (2016). Product stability and sequestration mechanisms in Solanum tuberosum engineered to biosynthesize high value ketocarotenoids. Plant Biotechnol J.

[CR161] Murata M, Nakai Y, Kawazu K, Ishizaka M, Kajiwara H, Abe H, Takeuchi K, Ichinose Y, Mitsuhara I, Mochizuki A, Seo S (2019). Loliolide, a Carotenoid Metabolite, Is a Potential Endogenous Inducer of Herbivore Resistance. Plant Physiol.

[CR162] Murchie EH, Ruban AV (2020). Dynamic non-photochemical quenching in plants: from molecular mechanism to productivity. Plant J.

[CR163] Neuman H, Galpaz N, Cunningham FX, Zamir D, Hirschberg J (2014). The tomato mutation nxd1 reveals a gene necessary for neoxanthin biosynthesis and demonstrates that violaxanthin is a sufficient precursor for abscisic acid biosynthesis. Plant J.

[CR164] Nisar N, Li L, Lu S, Khin NC, Pogson BJ (2015). Carotenoid metabolism in plants. Mol Plant.

[CR165] Niyogi KK, Truong TB (2013). Evolution of flexible non-photochemical quenching mechanisms that regulate light harvesting in oxygenic photosynthesis. Curr Opin Plant Biol.

[CR166] Nogueira M, Enfissi EMA, Welsch R, Beyer P, Zurbriggen MD, Fraser PD (2019). Construction of a fusion enzyme for astaxanthin formation and its characterisation in microbial and plant hosts: A new tool for engineering ketocarotenoids. Metab Eng.

[CR167] Ohmiya A, Kato M, Shimada T, Nashima K, Kishimoto S, Nagata M (2019). Molecular Basis of Carotenoid Accumulation in Horticultural Crops. Hortic J.

[CR168] Ohmiya A, Kishimoto S, Aida R, Yoshioka S, Sumitomo K (2006). Carotenoid cleavage dioxygenase (CmCCD4a) contributes to white color formation in chrysanthemum petals. Plant Physiol.

[CR169] Osorio CE (2019). The Role of Orange Gene in Carotenoid Accumulation: Manipulating Chromoplasts Toward a Colored Future. Front Plant Sci.

[CR170] Owen CR, Bradshaw HD (2011). Induced mutations affecting pollinator choice in Mimulus lewisii (Phrymaceae). Arthropod Plant Interact.

[CR171] Paine JA, Shipton CA, Chaggar S, Howells RM, Kennedy MJ, Vernon G, Wright SY, Hinchliffe E, Adams JL, Silverstone AL, Drake R (2005). Improving the nutritional value of Golden Rice through increased pro-vitamin A content. Nat Biotechnol.

[CR172] Pan X, Cao P, Su X, Liu Z, Li M (2020). Structural analysis and comparison of light-harvesting complexes I and II. Biochim Biophys Acta Bioenerg.

[CR173] Pan X, Li M, Wan T, Wang L, Jia C, Hou Z, Zhao X, Zhang J, Chang W (2011). Structural insights into energy regulation of light-harvesting complex CP29 from spinach. Nat Struct Mol Biol.

[CR174] Park H, Kreunen SS, Cuttriss AJ, DellaPenna D, Pogson BJ (2002). Identification of the carotenoid isomerase provides insight into carotenoid biosynthesis, prolamellar body formation, and photomorphogenesis. Plant Cell.

[CR175] Park S, Kim HS, Jung YJ, Kim SH, Ji CY, Wang Z, Jeong JC, Lee HS, Lee SY, Kwak SS (2016). Orange protein has a role in phytoene synthase stabilization in sweetpotato. Sci Rep.

[CR176] Park SY, Fung P, Nishimura N, Jensen DR, Fujii H, Zhao Y, Lumba S, Santiago J, Rodrigues A, Chow TF, Alfred SE, Bonetta D, Finkelstein R, Provart NJ, Desveaux D, Rodriguez PL, McCourt P, Zhu JK, Schroeder JI, Volkman BF, Cutler SR (2009). Abscisic acid inhibits type 2C protein phosphatases via the PYR/PYL family of START proteins. Science..

[CR177] Perreau F, Frey A, Effroy-Cuzzi D, Savane P, Berger A, Gissot L, Marion-Poll A (2020). ABSCISIC ACID-DEFICIENT4 has an essential function in both cis-violaxanthin and cis-neoxanthin synthesis. Plant Physiol.

[CR178] Pipitone R, Eicke S, Pfister B, Glauser G, Falconet D, Uwizeye C, Pralon T, Zeeman SC, Kessler F, Demarsy E (2021). A multifaceted analysis reveals two distinct phases of chloroplast biogenesis during de-etiolation in Arabidopsis. Elife..

[CR179] Proctor MS, Pazdernik M, Jackson PJ, Pilny J, Martin EC, Dickman MJ, Canniffe DP, Johnson MP, Hunter CN, Sobotka R, Hitchcock A (2020). Xanthophyll carotenoids stabilise the association of cyanobacterial chlorophyll synthase with the LHC-like protein HliD. Biochem J.

[CR180] Proust H, Hoffmann B, Xie X, Yoneyama K, Schaefer DG, Yoneyama K, Nogue F, Rameau C (2011). Strigolactones regulate protonema branching and act as a quorum sensing-like signal in the moss Physcomitrella patens. Development..

[CR181] Pulido P, Llamas E, Llorente B, Ventura S, Wright LP, Rodriguez-Concepcion M (2016). Specific Hsp100 Chaperones Determine the Fate of the First Enzyme of the Plastidial Isoprenoid Pathway for Either Refolding or Degradation by the Stromal Clp Protease in Arabidopsis. PLoS Genet.

[CR182] Pulido P, Toledo-Ortiz G, Phillips MA, Wright LP, Rodriguez-Concepcion M (2013). Arabidopsis J-protein J20 delivers the first enzyme of the plastidial isoprenoid pathway to protein quality control. Plant Cell.

[CR183] Qin X, Wang W, Chang L, Chen J, Wang P, Zhang J, He Y, Kuang T, Shen JR (2015). Isolation and characterization of a PSI-LHCI super-complex and its sub-complexes from a siphonaceous marine green alga, Bryopsis Corticulans. Photosynth Res.

[CR184] Quian-Ulloa R, Stange C (2021). Carotenoid Biosynthesis and Plastid Development in Plants: The Role of Light. Int J Mol Sci.

[CR185] Ramel F, Birtic S, Ginies C, Soubigou-Taconnat L, Triantaphylides C, Havaux M (2012). Carotenoid oxidation products are stress signals that mediate gene responses to singlet oxygen in plants. Proc Natl Acad Sci U S A.

[CR186] Ren C, Guo Y, Kong J, Lecourieux F, Dai Z, Li S, Liang Z (2020). Knockout of VvCCD8 gene in grapevine affects shoot branching. BMC Plant Biol.

[CR187] Rodrigo MJ, Lado J, Alós E, Alquézar B, Dery O, Hirschberg J, Zacarías L (2019). A mutant allele of ζ-carotene isomerase (Z-ISO) is associated with the yellow pigmentation of the “Pinalate” sweet orange mutant and reveals new insights into its role in fruit carotenogenesis. BMC Plant Biol.

[CR188] Rodriguez-Concepcion M, Avalos J, Bonet ML, Boronat A, Gomez-Gomez L, Hornero-Mendez D, Limon MC, Melendez-Martinez AJ, Olmedilla-Alonso B, Palou A, Ribot J, Rodrigo MJ, Zacarias L, Zhu C (2018). A global perspective on carotenoids: Metabolism, biotechnology, and benefits for nutrition and health. Prog Lipid Res.

[CR189] Rodriguez-Villalon A, Gas E, Rodriguez-Concepcion M (2009). Phytoene synthase activity controls the biosynthesis of carotenoids and the supply of their metabolic precursors in dark-grown Arabidopsis seedlings. Plant J.

[CR190] Ronen G, Carmel-Goren L, Zamir D, Hirschberg J (2000). An alternative pathway to beta -carotene formation in plant chromoplasts discovered by map-based cloning of beta and old-gold color mutations in tomato. Proc Natl Acad Sci U S A.

[CR191] Ronen G, Cohen M, Zamir D, Hirschberg J (1999). Regulation of carotenoid biosynthesis during tomato fruit development: expression of the gene for lycopene epsilon-cyclase is down-regulated during ripening and is elevated in the mutant Delta. Plant J.

[CR192] Ruban AV, Murchie EH (2012). Assessing the photoprotective effectiveness of non-photochemical chlorophyll fluorescence quenching: a new approach. Biochim Biophys Acta Bioenerg.

[CR193] Ruiz-Sola MA, Barja MV, Manzano D, Llorente B, Schipper B, Beekwilder J, Rodriguez-Concepcion M (2016). A Single Arabidopsis Gene Encodes Two Differentially Targeted Geranylgeranyl Diphosphate Synthase Isoforms. Plant Physiol.

[CR194] Ruiz-Sola MA, Rodriguez-Concepcion M (2012). Carotenoid biosynthesis in Arabidopsis: a colorful pathway. Arabidopsis Book.

[CR195] Sacharz J, Giovagnetti V, Ungerer P, Mastroianni G, Ruban AV (2017). The xanthophyll cycle affects reversible interactions between PsbS and light-harvesting complex II to control non-photochemical quenching. Nat Plants.

[CR196] Sadali NM, Sowden RG, Ling Q, Jarvis RP (2019). Differentiation of chromoplasts and other plastids in plants. Plant Cell Rep.

[CR197] Sagawa JM, Stanley LE, LaFountain AM, Frank HA, Liu C, Yuan YW (2016). An R2R3-MYB transcription factor regulates carotenoid pigmentation in Mimulus lewisii flowers. New Phytol.

[CR198] Sandmann G. Diversity and origin of carotenoid biosynthesis: its history of co-evolution towards plant photosynthesis. New Phytol. 2021. 10.1111/nph.17655. 10.1111/nph.17655 34324713

[CR199] Sauer L, Li B, Bernstein PS (2019). Ocular Carotenoid Status in Health and Disease. Annu Rev Nutr.

[CR200] Schaub P, Wuest F, Koschmieder J, Yu Q, Virk P, Tohme J, et al. Non-Enzymatic β-Carotene Degradation in (Provitamin A-Biofortified) Crop Plants. J Agric Food Chem. 2017. 10.1021/acs.jafc.7b01693. 10.1021/acs.jafc.7b01693 28703588

[CR201] Schwartz SH, Tan BC, Gage DA, Zeevaart JA, McCarty DR (1997). Specific oxidative cleavage of carotenoids by VP14 of maize. Science..

[CR202] Schweiggert R, Carle R (2017). Carotenoid deposition in plant and animal foods and its impact on bioavailability. Crit Rev Food Sci Nutr.

[CR203] Seto Y, Yasui R, Kameoka H, Tamiru M, Cao M, Terauchi R, Sakurada A, Hirano R, Kisugi T, Hanada A, Umehara M, Seo E, Akiyama K, Burke J, Takeda-Kamiya N, Li W, Hirano Y, Hakoshima T, Mashiguchi K, Noel JP, Kyozuka J, Yamaguchi S (2019). Strigolactone perception and deactivation by a hydrolase receptor DWARF14. Nat Commun.

[CR204] Shang Y, Venail J, Mackay S, Bailey PC, Schwinn KE, Jameson PE, Martin CR, Davies KM (2011). The molecular basis for venation patterning of pigmentation and its effect on pollinator attraction in flowers of Antirrhinum. New Phytol.

[CR205] Shumbe L, Bott R, Havaux M (2014). Dihydroactinidiolide, a high light-induced beta-carotene derivative that can regulate gene expression and photoacclimation in Arabidopsis. Mol Plant.

[CR206] Simpson K, Fuentes P, Quiroz-Iturra LF, Flores-Ortiz C, Contreras R, Handford M, Stange C (2018). Unraveling the induction of phytoene synthase 2 expression by salt stress and abscisic acid in Daucus carota. J Exp Bot.

[CR207] Son M, Pinnola A, Bassi R, Schlau-Cohen GS. Ultrabroadband two-dimensional electronic spectroscopy reveals energy flow pathways in LHCII across the visible spectrum: EPJ Web of Conferences: EDP Sciences; 2019. p. 09034. 10.1051/epjconf/201920509034

[CR208] Son M, Pinnola A, Gordon SC, Bassi R, Schlau-Cohen GS (2020). Observation of dissipative chlorophyll-to-carotenoid energy transfer in light-harvesting complex II in membrane nanodiscs. Nat Commun.

[CR209] Stanley L, Yuan YW (2019). Transcriptional Regulation of Carotenoid Biosynthesis in Plants: So Many Regulators, So Little Consensus. Front Plant Sci.

[CR210] Stanley LE, Ding B, Sun W, Mou F, Hill C, Chen S, Yuan YW (2020). A Tetratricopeptide Repeat Protein Regulates Carotenoid Biosynthesis and Chromoplast Development in Monkeyflowers (Mimulus). Plant Cell.

[CR211] Sun T, Li L (2020). Toward the ‘golden’ era: The status in uncovering the regulatory control of carotenoid accumulation in plants. Plant Sci.

[CR212] Sun T, Tadmor Y, Li L. Pathways for carotenoid biosynthesis, degradation, and storage. Plant and Food Carotenoids: Springer; 2020a. p. 3–23. 10.1007/978-1-4939-9952-1_1 31745909

[CR213] Sun T, Yuan H, Cao H, Yazdani M, Tadmor Y, Li L (2018). Carotenoid Metabolism in Plants: The Role of Plastids. Mol Plant.

[CR214] Sun T, Yuan H, Chen C, Kadirjan-Kalbach DK, Mazourek M, Osteryoung KW, Li L (2020). OR (His), a Natural Variant of OR, Specifically Interacts with Plastid Division Factor ARC3 to Regulate Chromoplast Number and Carotenoid Accumulation. Mol Plant.

[CR215] Sun T, Zhou F, Huang XQ, Chen WC, Kong MJ, Zhou CF, Zhuang Z, Li L, Lu S (2019). ORANGE Represses Chloroplast Biogenesis in Etiolated Arabidopsis Cotyledons via Interaction with TCP14. Plant Cell.

[CR216] Sun T, Zhu Q, Wei Z, Owens LA, Fish T, Kim H, Thannhauser TW, Cahoon EB, Li L (2021). Multi-strategy engineering greatly enhances provitamin A carotenoid accumulation and stability in Arabidopsis seeds. aBIOTECH.

[CR217] Takaichi S (2011). Carotenoids in algae: distributions, biosyntheses and functions. Mar Drugs.

[CR218] Tan BC, Joseph LM, Deng WT, Liu L, Li QB, Cline K, McCarty DR (2003). Molecular characterization of the Arabidopsis 9-cis epoxycarotenoid dioxygenase gene family. Plant J.

[CR219] Tata SK, Jung J, Kim YH, Choi JY, Jung JY, Lee IJ, Shin JS, Ryu SB (2016). Heterologous expression of chloroplast-localized geranylgeranyl pyrophosphate synthase confers fast plant growth, early flowering and increased seed yield. Plant Biotechnol J.

[CR220] Telfer A (2005). Too much light? How β-carotene protects the photosystem II reaction centre. Photochem Photobiol Sci.

[CR221] Toledo-Ortiz G, Huq E, Rodriguez-Concepcion M (2010). Direct regulation of phytoene synthase gene expression and carotenoid biosynthesis by phytochrome-interacting factors. Proc Natl Acad Sci U S A.

[CR222] Toledo-Ortiz G, Johansson H, Lee KP, Bou-Torrent J, Stewart K, Steel G, Rodriguez-Concepcion M, Halliday KJ (2014). The HY5-PIF regulatory module coordinates light and temperature control of photosynthetic gene transcription. PLoS Genet.

[CR223] Torres-Montilla S, Rodriguez-Concepcion M (2021). Making extra room for carotenoids in plant cells: New opportunities for biofortification. Prog Lipid Res.

[CR224] Tzuri G, Zhou X, Chayut N, Yuan H, Portnoy V, Meir A, Sa'ar U, Baumkoler F, Mazourek M, Lewinsohn E (2015). A ‘golden’ SNP in CmOr governs the fruit flesh color of melon (C ucumis melo). Plant J.

[CR225] Umena Y, Kawakami K, Shen JR, Kamiya N (2011). Crystal structure of oxygen-evolving photosystem II at a resolution of 1.9 angstrom. Nature..

[CR226] Valenta K, Kalbitzer U, Razafimandimby D, Omeja P, Ayasse M, Chapman CA, Nevo O (2018). The evolution of fruit colour: phylogeny, abiotic factors and the role of mutualists. Sci Rep.

[CR227] Vogel JT, Tan BC, McCarty DR, Klee HJ (2008). The carotenoid cleavage dioxygenase 1 enzyme has broad substrate specificity, cleaving multiple carotenoids at two different bond positions. J Biol Chem.

[CR228] Wang B, Luo Q, Li Y, Yin L, Zhou N, Li X, Gan J, Dong A (2020). Structural insights into target DNA recognition by R2R3-MYB transcription factors. Nucleic Acids Res.

[CR229] Wang JY, Haider I, Jamil M, Fiorilli V, Saito Y, Mi J, Baz L, Kountche BA, Jia KP, Guo X, Balakrishna A, Ntui VO, Reinke B, Volpe V, Gojobori T, Blilou I, Lanfranco L, Bonfante P, Al-Babili S (2019). The apocarotenoid metabolite zaxinone regulates growth and strigolactone biosynthesis in rice. Nat Commun.

[CR230] Wang JY, Lin P-Y, Al-Babili S. On the biosynthesis and evolution of apocarotenoid plant growth regulators. Semin Cell Dev Biol. 2021a:3–11. Elsevier. 10.1016/j.semcdb.2020.07.007. 10.1016/j.semcdb.2020.07.007 32732130

[CR231] Wang P, Wang Y, Wang W, Chen T, Tian S, Qin G (2020). Ubiquitination of phytoene synthase 1 precursor modulates carotenoid biosynthesis in tomato. Commun Biol.

[CR232] Wang W, Wang P, Li X, Wang Y, Tian S, Qin G (2021). The transcription factor SlHY5 regulates the ripening of tomato fruit at both the transcriptional and translational levels. Hortic Res.

[CR233] Waters MT, Gutjahr C, Bennett T, Nelson DC (2017). Strigolactone Signaling and Evolution. Annu Rev Plant Biol.

[CR234] Watkins JL, Pogson BJ (2020). Prospects for Carotenoid Biofortification Targeting Retention and Catabolism. Trends Plant Sci.

[CR235] Wei S, Hannoufa A, Soroka J, Xu N, Li X, Zebarjadi A, Gruber M (2011). Enhanced beta-ionone emission in Arabidopsis over-expressing AtCCD1 reduces feeding damage in vivo by the crucifer flea beetle. Environ Entomol.

[CR236] Welsch R, Beyer P, Hugueney P, Kleinig H, von Lintig J (2000). Regulation and activation of phytoene synthase, a key enzyme in carotenoid biosynthesis, during photomorphogenesis. Planta..

[CR237] Welsch R, Zhou X, Yuan H, Alvarez D, Sun T, Schlossarek D, Yang Y, Shen G, Zhang H, Rodriguez-Concepcion M, Thannhauser TW, Li L (2018). Clp Protease and OR Directly Control the Proteostasis of Phytoene Synthase, the Crucial Enzyme for Carotenoid Biosynthesis in Arabidopsis. Mol Plant.

[CR238] Wen X, Heller A, Wang K, Han Q, Ni Y, Carle R, Schweiggert R (2020). Carotenogenesis and chromoplast development during ripening of yellow, orange and red colored Physalis fruit. Planta..

[CR239] Wilk L, Grunwald M, Liao P-N, Walla PJ, Kühlbrandt W (2013). Direct interaction of the major light-harvesting complex II and PsbS in nonphotochemical quenching. Proc Natl Acad Sci U S A.

[CR240] Wright LP, Rohwer JM, Ghirardo A, Hammerbacher A, Ortiz-Alcaide M, Raguschke B, Schnitzler JP, Gershenzon J, Phillips MA (2014). Deoxyxylulose 5-Phosphate Synthase Controls Flux through the Methylerythritol 4-Phosphate Pathway in Arabidopsis. Plant Physiol.

[CR241] Wurtzel ET (2019). Changing Form and Function through Carotenoids and Synthetic Biology. Plant Physiol.

[CR242] Wurtzel ET, Cuttriss A, Vallabhaneni R (2012). Maize provitamin a carotenoids, current resources, and future metabolic engineering challenges. Front Plant Sci.

[CR243] Xiao K, Chen J, He Q, Wang Y, Shen H, Sun L (2020). DNA methylation is involved in the regulation of pepper fruit ripening and interacts with phytohormones. J Exp Bot.

[CR244] Xu P, Chukhutsina VU, Nawrocki WJ, Schansker G, Bielczynski LW, Lu Y, Karcher D, Bock R, Croce R (2020). Photosynthesis without β-carotene. Elife..

[CR245] Yan J, Kandianis CB, Harjes CE, Bai L, Kim EH, Yang X, Skinner DJ, Fu Z, Mitchell S, Li Q, Fernandez MG, Zaharieva M, Babu R, Fu Y, Palacios N, Li J, Dellapenna D, Brutnell T, Buckler ES, Warburton ML, Rocheford T (2010). Rare genetic variation at Zea mays crtRB1 increases beta-carotene in maize grain. Nat Genet.

[CR246] Yazdani M, Sun Z, Yuan H, Zeng S, Thannhauser TW, Vrebalov J, Ma Q, Xu Y, Fei Z, Van Eck J, Tian S, Tadmor Y, Giovannoni JJ, Li L (2019). Ectopic expression of ORANGE promotes carotenoid accumulation and fruit development in tomato. Plant Biotechnol J.

[CR247] Yuan H, Owsiany K, Sheeja TE, Zhou X, Rodriguez C, Li Y, Welsch R, Chayut N, Yang Y, Thannhauser TW, Parthasarathy MV, Xu Q, Deng X, Fei Z, Schaffer A, Katzir N, Burger J, Tadmor Y, Li L (2015). A Single Amino Acid Substitution in an ORANGE Protein Promotes Carotenoid Overaccumulation in Arabidopsis. Plant Physiol.

[CR248] Yuan H, Pawlowski EG, Yang Y, Sun T, Thannhauser TW, Mazourek M, Schnell D, Li L (2021). Arabidopsis ORANGE protein regulates plastid pre-protein import through interacting with Tic proteins. J Exp Bot.

[CR249] Yuan H, Zhang J, Nageswaran D, Li L (2015). Carotenoid metabolism and regulation in horticultural crops. Hortic Res.

[CR250] Yuan YW, Byers KJ, Bradshaw HD (2013). The genetic control of flower-pollinator specificity. Curr Opin Plant Biol.

[CR251] Zhang J, Sun H, Guo S, Ren Y, Li M, Wang J, Zhang H, Gong G, Xu Y (2020). Decreased Protein Abundance of Lycopene beta-Cyclase Contributes to Red Flesh in Domesticated Watermelon. Plant Physiol.

[CR252] Zhang J, Yuan H, Fei Z, Pogson BJ, Zhang L, Li L (2015). Molecular characterization and transcriptome analysis of orange head Chinese cabbage (Brassica rapa L. ssp. pekinensis). Planta..

[CR253] Zheng X, Giuliano G, Al-Babili S (2020). Carotenoid biofortification in crop plants: citius, altius, fortius. Biochim Biophys Acta Mol Cell Biol Lipids.

[CR254] Zheng X, Zhu K, Sun Q, Zhang W, Wang X, Cao H, Tan M, Xie Z, Zeng Y, Ye J, Chai L, Xu Q, Pan Z, Xiao S, Fraser PD, Deng X (2019). Natural Variation in CCD4 Promoter Underpins Species-Specific Evolution of Red Coloration in Citrus Peel. Mol Plant.

[CR255] Zhong S, Fei Z, Chen YR, Zheng Y, Huang M, Vrebalov J, McQuinn R, Gapper N, Liu B, Xiang J, Shao Y, Giovannoni JJ (2013). Single-base resolution methylomes of tomato fruit development reveal epigenome modifications associated with ripening. Nat Biotechnol.

[CR256] Zhong Y, Pan X, Wang R, Xu J, Guo J, Yang T, Zhao J, Nadeem F, Liu X, Shan H, Xu Y, Li X (2020). ZmCCD10a Encodes a Distinct Type of Carotenoid Cleavage Dioxygenase and Enhances Plant Tolerance to Low Phosphate. Plant Physiol.

[CR257] Zhou D, Shen Y, Zhou P, Fatima M, Lin J, Yue J, Zhang X, Chen LY, Ming R (2019). Papaya CpbHLH1/2 regulate carotenoid biosynthesis-related genes during papaya fruit ripening. Hortic Res.

[CR258] Zhou F, Lin Q, Zhu L, Ren Y, Zhou K, Shabek N, Wu F, Mao H, Dong W, Gan L, Ma W, Gao H, Chen J, Yang C, Wang D, Tan J, Zhang X, Guo X, Wang J, Jiang L, Liu X, Chen W, Chu J, Yan C, Ueno K, Ito S, Asami T, Cheng Z, Wang J, Lei C, Zhai H, Wu C, Wang H, Zheng N, Wan J (2013). D14-SCF(D3)-dependent degradation of D53 regulates strigolactone signalling. Nature..

[CR259] Zhou F, Wang CY, Gutensohn M, Jiang L, Zhang P, Zhang D, Dudareva N, Lu S (2017). A recruiting protein of geranylgeranyl diphosphate synthase controls metabolic flux toward chlorophyll biosynthesis in rice. Proc Natl Acad Sci U S A.

[CR260] Zhou H, Yang M, Zhao L, Zhu Z, Liu F, Sun H, Sun C, Tan L (2021). HIGH-TILLERING AND DWARF 12 modulates photosynthesis and plant architecture by affecting carotenoid biosynthesis in rice. J Exp Bot.

[CR261] Zhou J, Zeng L, Liu J, Xing D (2015). Manipulation of the Xanthophyll Cycle Increases Plant Susceptibility to Sclerotinia sclerotiorum. PLoS Pathog.

[CR262] Zhou X, Sun TH, Wang N, Ling HQ, Lu S, Li L (2011). The cauliflower *Orange* gene enhances petiole elongation by suppressing expression of *eukaryotic release factor 1*. New Phytol.

[CR263] Zhou X, Welsch R, Yang Y, Alvarez D, Riediger M, Yuan H, Fish T, Liu J, Thannhauser TW, Li L (2015). Arabidopsis OR proteins are the major posttranscriptional regulators of phytoene synthase in controlling carotenoid biosynthesis. Proc Natl Acad Sci U S A.

[CR264] Zhu K, Sun Q, Chen H, Mei X, Lu S, Ye J, Chai L, Xu Q, Deng X (2021). Ethylene activation of carotenoid biosynthesis by a novel transcription factor CsERF061. J Exp Bot.

[CR265] Zhu K, Zheng X, Ye J, Huang Y, Chen H, Mei X, Xie Z, Cao L, Zeng Y, Larkin RM (2021). Regulation of carotenoid and chlorophyll pools in hesperidia, anatomically unique fruits found only in Citrus. Plant Physiol.

